# Assessing the consistency assumptions underlying network meta‐regression using aggregate data

**DOI:** 10.1002/jrsm.1327

**Published:** 2018-11-12

**Authors:** Sarah Donegan, Sofia Dias, Nicky J. Welton

**Affiliations:** ^1^ Department of Biostatistics, Waterhouse Building University of Liverpool Liverpool UK; ^2^ School of Social and Community Medicine University of Bristol Bristol UK

**Keywords:** consistency, inconsistency models, network meta‐analysis, network meta‐regression, node splitting, treatment by covariate interactions

## Abstract

When numerous treatments exist for a disease (Treatments 1, 2, 3, etc), network meta‐regression (NMR) examines whether each relative treatment effect (eg, mean difference for 2 vs 1, 3 vs 1, and 3 vs 2) differs according to a covariate (eg, disease severity). Two consistency assumptions underlie NMR: consistency of the treatment effects at the covariate value 0 and consistency of the regression coefficients for the treatment by covariate interaction. The NMR results may be unreliable when the assumptions do not hold. Furthermore, interactions may exist but are not found because inconsistency of the coefficients is masking them, for example, when the treatment effect increases as the covariate increases using direct evidence but the effect decreases with the increasing covariate using indirect evidence.

We outline existing NMR models that incorporate different types of treatment by covariate interaction. We then introduce models that can be used to assess the consistency assumptions underlying NMR for aggregate data. We extend existing node‐splitting models, the unrelated mean effects inconsistency model, and the design by treatment inconsistency model to incorporate covariate interactions. We propose models for assessing both consistency assumptions simultaneously and models for assessing each of the assumptions in turn to gain a more thorough understanding of consistency.

We apply the methods in a Bayesian framework to trial‐level data comparing antimalarial treatments using the covariate average age and to four fabricated data sets to demonstrate key scenarios.

We discuss the pros and cons of the methods and important considerations when applying models to aggregated data.

## 1 INTRODUCTION

Reviews often compare multiple treatments for the same condition. In such cases, network meta‐analysis (NMA) can compare all treatments (eg, Treatments 1, 2, and 3*)* in a single analysis by estimating the relative treatment effects (eg, log odds ratios) for all treatment pairings (eg, 2 vs 1, 3 vs 1, and 3 vs 2) using direct and indirect evidence.^1^, ^2^, ^3^ The key assumption underlying NMA is consistency of the treatments effects across direct and indirect evidence.^3^ Many methods have been proposed to assess the consistency assumption underlying NMA,^4^ including node‐splitting models^5^, ^6^ and inconsistency models, such as the design by treatment (DBT) inconsistency model^7^, ^8^, ^9^, ^10^, ^11^ and the unrelated mean effects (URM) inconsistency model.^12^

Network meta‐regression (NMR) is an extension of NMA that examines whether a covariate modifies each of the relative treatment effects.^13^ A covariate may modify each relative treatment effect differently; that is, each treatment comparison may have a different covariate interaction. NMR is used to explore causes of heterogeneity or inconsistency or when known effect modifiers exist and we wish to present results for different patient groups. Covariates may be characteristics of patients (eg, weight), treatments (eg, additional therapy), studies (eg, location), or methods (eg, allocation concealment).^14^, ^15^, ^16^

NMR results commonly consist of, for each comparison, one relative treatment effect estimated at the covariate value 0 (or at the mean covariate value when the NMR model is centered) and one regression coefficient for the treatment by covariate interaction. Consistency assumptions are required for both of these parameters.^17^, ^18^, ^19^ For instance, for a three‐treatment NMR, where Treatment 1 is taken as the reference, the consistency equation for the relative treatment effects can be written as, *d*_23_ = *d*_13_ − *d*_12_ where, for example, *d*_23_ is the relative treatment effect for 3 vs 2, and the consistency equation for the regression coefficients is *β*_23_ = *β*_13_ − *β*_12_ where, for example, *β*_23_ is the coefficient for 3 vs 2.^13^, ^17^, ^19^ It is possible for neither assumption to hold (ie, inconsistent relative treatment effects and inconsistent coefficients) or for only one of the assumptions to hold (ie, either consistent relative treatment effects or consistent coefficients), which would make the results of the NMR unreliable.

**Figure 1:**
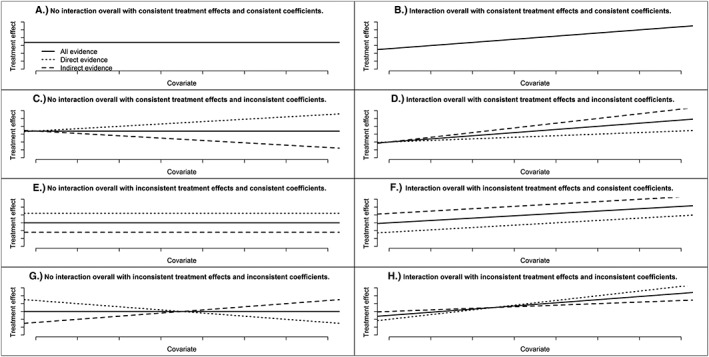
Graphs showing how the relative treatment effect (eg, log odds ratio) for Treatment 3 vs Treatment 2 could change with a covariate value with separate lines representing direct evidence (from trials that allocated Treatments 2 and 3), indirect evidence (from the remaining trials), and all evidence in various scenarios: A, there is no treatment by covariate interaction based on all evidence, and the relative treatment effects at 0 covariate are consistent, and the regression coefficients for the treatment by covariate interaction are consistent; B, there is an interaction based on all evidence, and the relative treatment effects at 0 covariate are consistent, and the coefficients are consistent; C, there is no interaction based on all evidence, and the relative treatment effects at 0 covariate are consistent, and the coefficients are inconsistent; D, there is an interaction based on all evidence, and the relative treatment effects at 0 covariate are consistent, and the coefficients are inconsistent; E, there is no interaction based on all evidence, and the relative treatment effects at 0 covariate are inconsistent, and the coefficients are consistent; F, there is an interaction based on all evidence, and the relative treatment effects at 0 covariate are inconsistent, and the coefficients are consistent; G, there is no interaction based on all evidence, and the relative treatment effects at 0 covariate are inconsistent, and the coefficients are inconsistent; and H, there is an interaction based on all evidence, and the relative treatment effects at 0 covariate are inconsistent, and the coefficients are inconsistent. Direct, indirect, and all evidence is overlapping in plots (A) and (B)

Theoretically, there are eight possible scenarios that can occur when assessing whether treatment by covariate interactions exist and the consistency assumptions. Examples of the scenarios are shown in Figure 1A‐H. Each figure shows how the relative treatment effect for 3 vs 2 changes with an increasing covariate value; separate lines are displayed for direct, indirect, and all evidence. For a three‐treatment network, the direct evidence for 3 vs 2 would be from trials that allocated Treatments 2 and 3 and the indirect evidence for 3 vs 2 would be from the remaining trials. Note that the lines have the same intercept when the relative treatment effects at the covariate value 0 are consistent (Figure 1A‐D) and the lines have the same slope when the coefficients are consistent (Figure 1A, B, E, and F). In Figure 1A, no interaction is detected using NMR, and both consistency assumptions are satisfied; therefore, the NMR results are valid but would not be clinically useful. On the other hand, in Figure 1B, NMR shows an interaction and both assumptions hold; therefore, the NMR is reliable and could be used to draw clinical inferences. Figure 1C, E, and G show scenarios where no interaction is detected using NMR, but one or more of the assumptions are not satisfied; consequently, the NMR results are invalid; notably, in Figure 1C,G, an interaction exists when direct evidence, and indirect evidence are considered separately, but it is not seen when applying NMR because it is masked by the inconsistency. Lastly, in Figure 1D, F, and H, an interaction is found using NMR, but one or more of the assumptions do not hold, so the NMR results are unreliable. The cause of inconsistency should be considered when inconsistency is found (Figures 1C‐H).

Although many methodological publications have proposed NMR analyses,^13^, ^17^, ^18^, ^19^, ^20^, ^21^, ^22^, ^23^, ^24^, ^25^ to the authors' knowledge, no methods have been introduced for assessing the consistency assumptions underlying NMR.

In this paper, we introduce methods for assessing the consistency assumptions underlying NMR. We extend existing node‐splitting models,^5^, ^6^ the DBT inconsistency model,^7^, ^8^, ^9^, ^10^, ^11^ and the URM inconsistency model^12^ to incorporate treatment by covariate interactions. In Section 2, we specify the NMR model and propose assessment methods that can be applied to aggregate trial‐level data (ie, trial specific relative treatment effects relative to reference Arm 1 and their variances) with either continuous or categorical covariates. In Section 3, we apply the methods to a real data set and fabricated data sets illustrating key scenarios under a Bayesian framework. In Section 4, we discuss the proposed methods and highlight their pros and cons.

## 2 METHODS

We outline NMR models and then introduce methods for assessing consistency using the node‐splitting models and one type of inconsistency model (ie, URM model). New methods based on the alternative DBT inconsistency model are also presented in the supplementary material. All models are summarized in Table 1.

**Table 1:** Proposed model variations

		**Models Including Independent Treatment by Covariate Interactions**	**Models Including Exchangeable Treatment by Covariate Interactions**	**Models Including Common Treatment by Covariate Interactions**
**NMR Models**		Model 1a	Model 1b	Model 1c
**Node‐splitting models**	**Models splitting the relative treatment effect and the regression coefficient for the interaction.**	Model 2.1a	Model 2.1b	Model 2.1c
	**Models splitting the relative treatment effect only.**	Model 2.2a	Model 2.2b	Model 2.2c
	**Models splitting the regression coefficient for the interaction only.**	Model 2.3a	Model 2.3b	Model 2.3c
**URM models**	**Models assessing consistency of the relative treatment effect and the regression coefficient for the interaction.**	Model 3.1a	Model 3.1b	Model 3.1c
	**Models assessing consistency of the relative treatment effect only.**	Model 3.2a	Model 3.2b	Model 3.2c
	**Models assessing consistency of the regression coefficient for the interaction only.**	Model 3.3a	Model 3.3b	Model 3.3c
**DBT models**	**Models assessing consistency of the relative treatment effect and the regression coefficient for the interaction.**	Model 4.1a	Model 4.1b	Model 4.1c
	**Models assessing consistency of the relative treatment effect only.**	Model 4.2a	Model 4.2b	Model 4.2c
	**Models assessing consistency of the regression coefficient for the interaction only.**	Model 4.3a	Model 4.3b	Model 4.3c

Abbreviations: DBT, design by treatment; NMR, network meta‐regression; URM, unrelated mean effects.

To set notation, let *i* denote the trial where *i* = 1, …, *S* and *S* is the number of independent trials and let *k* be the trial arm where *k* = 1, …, *A*_*i*_ and *A*_*i*_ is the number of arms in trial *i*. Let *t*_*ik*_ denote the treatment given in trial *i* in arm *k* where *t*_*ik*_ ∈ {1, ……, *T*} and *T* is the number of treatments in the network. Note that Treatment 1 is taken to be the reference treatment.

Suppose we have trial‐level outcome data, where *y*_*ik*_ is the observed relative treatment effect (eg, log odds ratio or mean difference) for arm *k* vs Arm 1 (with *k* ≥ 2) in trial *i* and *v*_*ik*_ is the corresponding variance. As the relative treatment effect is a continuous measure, we assume a normal likelihood *y*_*ik*_~*N*(*θ*_*ik*,_*v*_*ik*_) where *θ*_*ik*_ is the mean relative treatment effect in trial *i* (with *k* ≥ 2). Also, the data set would include a study‐level covariate *x*_*i*_ for each trial *i* that can be a continuous variable or an indicator variable to represent dichotomous data.

### 2.1 Network meta‐regression models

NMR models estimate the basic regression coefficients, which are the coefficients for each treatment vs Treatment 1 (ie, *β*_12_, *β*_13_, …, *β*_1*T*_), and then the remaining functional coefficients (ie, *β*_23_, *β*_24_, ….) are calculated as linear combinations of the basic coefficients using the consistency equations. Three NMR models have been proposed previously, each making different assumptions regarding the basic coefficients,^13^, ^17^, ^18^, ^19^ that is, independent (model 1a), exchangeable (model 1b), and common coefficients (model 1c). The decision regarding which assumption to make can be based on model fit statistics and the estimated coefficients of the models but in practice is often determined by data availability.

Model 1a can be written as follows:

$$\theta_{\mathrm{ik}}=\delta_{i,1k}+\beta_{t_{i1},t_{\mathrm{ik}}}x_{i},$$

Where $\beta_{t_{i1},t_{\mathrm{ik}}}$= $\beta_{1,t_{\mathrm{ik}}}$‐ $\beta_{1,t_{i1}}$, $\beta_{t_{i1},t_{\mathrm{ik}}}$ is the difference in the relative treatment effect of *t*_*ik*_ vs *t*_*i*1_ per unit increase in the covariate *x*_*i*_, or in other words, the regression coefficient for the treatment by covariate interaction. In a random‐effects model, *δ*_*i*,1*k*_ (with *k* ≥ 2) represents the trial‐specific relative treatment effect of *t*_*ik*_ vs *t*_*i*1_ when the covariate is 0 (*x*_*i*_ = 0) and is assumed to be a realization from a normal distribution $\delta_{i,1k}∼N\left(d_{t_{i1},t_{\mathrm{ik}}},\sigma^{2}\right)$ with $d_{t_{i1},t_{\mathrm{ik}}}=d_{1,t_{\mathrm{ik}}}-d_{1,t_{i1}}$ where $d_{t_{i1},t_{\mathrm{ik}}}$ is the mean relative treatment effect of *t*_*ik*_ vs *t*_*i*1_ when the covariate is 0. In a fixed‐effect model, we set *σ*^2^ = 0 to obtain *δ*_*i*,1*k*_= $d_{1,t_{\mathrm{ik}}}-d_{1,t_{i1}}$.

Model 1b is the same as model 1a, but now, $\beta_{1,t_{\mathrm{ik}}}∼\text{Norm}\left(B,\upsilon^{2}\right).$ Model 1c is formulated by setting $\beta_{1,t_{\mathrm{ik}}}=\beta$ in model 1a; note that in this model, the functional coefficients are 0 because of the consistency equations (eg, *β*_23_ = *β*_13_ − *β*_12_ = *β* − *β* = 0).^17^

### 2.2 Assessing consistency by node splitting

The principle aim of node‐splitting models is to assess whether there is evidence of “loop inconsistency,” where loop inconsistency is defined as a difference between a result from direct and indirect evidence. Node‐splitting models estimate relative treatment effects and/or regression coefficients for the interaction based on direct evidence and separate estimates from indirect evidence to explore whether they agree. Multiple node‐splitting models need to be applied, one model for each comparison of interest.

To specify the node‐splitting models, we extend the notation, such that the node being split is ( $\hat{t}$, t^*^) where $\hat{t}\neq t^{*}\;$and $\hat{t}<t^{*}.$For example, if one wants to split the node (*3*, *4*), then $\hat{t}=3$ and t^*^ = 4.

To assess both the consistency assumptions simultaneously, node‐splitting models can split the relative treatment effect and coefficient to provide, for each comparison with both direct and indirect evidence, a relative treatment effect, and a coefficient estimated from direct evidence and an effect and coefficient based on indirect evidence. The model that splits the relative treatment effect and coefficient and includes independent interactions (model 2.1a) is an extension of model 1a as follows:

θik=δi,1k+βti1,tikxiifti1≠t^and/ortik≠t*δi,1k+βdirxiifti1=t^andtik=t*,

Where $\beta_{t_{i1},t_{\mathrm{ik}}}$= $\beta_{1,t_{\mathrm{ik}}}$‐ $\beta_{1,t_{i1}}$, $\;\beta_{t_{i1},t_{\mathrm{ik}}}$ represents the difference in the relative treatment effect of *t*_*ik*_ vs *t*_*i*1_ per unit increase in the covariate estimated using indirect evidence, and *β*^*dir*^ represents the difference in the relative treatment effect of t^*^ vs $\hat{t}$ per unit increase in the covariate estimated using direct evidence. In a random‐effects model, if trial *i* allocated t^*^and $\hat{t}$, that is, *t*_*i*1_= $\hat{t}$ and *t*_*ik*_ = t^*^, then *δ*_*i*,1*k*_~*N*(*d*^*dir*^, *σ*^2^) where *d*^*dir*^ represents the mean relative treatment effect of $t^{*}\;\mathrm{vs}\;\hat{t}$ when the covariate value is 0 estimated using direct evidence; whereas if trial *i* did not allocate t^*^and $\hat{t}$, that is, *t*_*i*1_≠ $\hat{t}$ and/or *t*_*ik*_ ≠ t^*^, then $\delta_{i,1k}∼N\left(d_{t_{i1},t_{\mathrm{ik}}},\sigma^{2}\right)\;$where $d_{t_{i1},t_{\mathrm{ik}}}$ represents the mean relative treatment effect of *t*_*ik*_ vs *t*_*i*1_ when the covariate value is 0 estimated using indirect evidence and $d_{t_{i1},t_{\mathrm{ik}}}=d_{1,t_{\mathrm{ik}}}-d_{1,t_{i1}}$.

To assess only the consistency of the relative treatment effects, node‐splitting models can split the relative treatment effect alone to produce a single coefficient that is estimated using all evidence and two relative treatment effects (ie, one estimated using direct evidence and the other estimated using the indirect evidence). The model that splits the relative treatment effect alone and includes independent interactions (model 2.2a) is

$$\theta_{\mathrm{ik}}=\delta_{i,1k}+\beta_{t_{i1},t_{\mathrm{ik}}}x_{i},$$

where $\beta_{t_{i1},t_{\mathrm{ik}}}$ represents the difference in the relative treatment effect of *t*_*ik*_ vs. *t*_*i*1_ per unit increase in the covariate estimated using all evidence. In this model, the trial‐specific relative treatment effects, *δ*_*i*,1*k*_ are distributed in the same way as in model 2.1a.

Likewise, to assess the consistency of the coefficients alone, a node‐splitting model can split only the coefficient to estimate a single relative treatment effect using all evidence and two coefficients (e, one estimated from direct evidence and the other from indirect evidence). The model that splits only the coefficient and includes independent interactions (model 2.3a) is the same as model 2.1a except the trial‐specific relative treatment effects; *δ*_*i*,1*k*_ are distributed as $\delta_{i,1k}∼N\left(d_{t_{i1},t_{\mathrm{ik}}},\sigma^{2}\right)\;$where $\;d_{t_{i1},t_{\mathrm{ik}}}$ represents the mean relative treatment effect of *t*_*ik*_ vs *t*_*i*1_ when the covariate value is 0 estimated using all evidence.

Node‐splitting models can be adapted to include exchangeable (models 2.1b, 2.2b, and 2.3b) or common (models 2.1c, 2.2c, and 2.3c) interactions as described in Section 2.1. Note that model 2.1c and 2.3c fix each functional coefficient based on indirect evidence (ie, $\beta_{t_{i1},t_{\mathrm{ik}}}$when *t*_*i*1_ ≠ 1) to be 0 whereas the corresponding result from direct evidence (*β*^*dir*^) is not.

The level of consistency can be assessed, by comparing the model fit of the NMR (model 1[a, b, or c]) with that of the node‐splitting models (models 2.1[a, b, or c], 2.2[a, b, or c], and *2.3*[a, b, or c]); inconsistency is indicated if a node‐splitting model is an improved fit. Moreover, if the between trial variance is lower in the node‐splitting models as compared with the NMR, inconsistency may exist. Also, for each treatment comparison, the size, direction, and precision of the relative treatment effect estimated using direct evidence can be compared with that estimated using indirect evidence. Such comparisons are subjective and when results are presented graphically and compared, care must be taken because the scale and shape of the plots can affect how different the results appear to be. Furthermore, when using Bayesian methods, for each comparison, the probability (prob) that the direct and indirect evidence differs can be calculated. For each treatment pairing, the inconsistency estimate (IE); that is, the difference between the relative treatment effect from direct evidence and indirect evidence can be calculated at each iteration of the chain, and the number of iterations for which *IE* ≥ 0 is counted. It is then possible to calculate the prob that the relative treatment effect from direct evidence exceeds the relative treatment effect from indirect evidence, by dividing the number of counted iterations by the total number of iterations of the chain. Lastly, assuming that the posterior distribution of the difference (IE) is symmetric and unimodal, the prob that the direct and indirect evidence agree is given by *P* = 2 × *minimum*(*prob*, 1 − *prob*). 5, 26 Likewise, the regression coefficients from direct and indirect evidence can be compared in the same way.

### 2.3 Assessing consistency using URM models

URM models assess global consistency that is inconsistency somewhere in the treatment network, by comparing the results from an NMR model with those from an URM model.^12^

The URM model that assesses the consistency of the relative treatment effects and coefficients and includes independent interactions (model 3.1a) is the same as the NMR model (model 1a), but it does not incorporate the consistency equations (i.e. $d_{t_{i1},t_{\mathrm{ik}}}=d_{1,t_{\mathrm{ik}}}-d_{1,t_{i1}}$ and $\beta_{t_{i1},t_{\mathrm{ik}}}$= $\beta_{1,t_{\mathrm{ik}}}$‐ $\beta_{1,t_{i1}}$), and as such, the model parameters are estimated using direct evidence only. Model 3.1a is equivalent to fitting separate pair‐wise meta‐regressions, except, *model 3.1a* assumes the between trial variance (*σ*^2^) is equal across comparisons but the pair‐wise meta‐regressions would not.

The URM model that assesses only consistency of the relative treatment effects and includes independent interactions (model 3.2a) is the same as model 3.1a but incorporates the consistency equation for the coefficients. Likewise, the UMR model that assesses only consistency of the coefficients with independent interactions (model 3.3a) is same as model 3.1a but includes the consistency equation for the relative treatment effects.

Exchangeable (models 3.1b, 3.2b, and 3.3b) or common (models 3.1c, 3.2c, and 3.3c) interactions can be included. However, it is worth noting that the independent, exchangeable, or common assumptions are slightly different to those specified for the NMR models (models 1a, 1b, and 1c). In the NMR models, we assume the basic regression coefficients (ie, *β*_12_, *β*_13_, …, *β*_1*T*_) are independent, exchangeable, or common. However, when the consistency equation for the coefficients is not used in the URM model (ie, models 3.1[a, b, or c] and 3.3[a, b, or c) we can assume that all regression coefficients, that is basic and functional coefficients, are independent, exchangeable (ie, $\beta_{t_{i1},t_{\mathrm{ik}}}∼\text{Norm}\left(B,\upsilon^{2}\right)$) or common (ie, $\beta_{t_{i1},t_{\mathrm{ik}}}=\beta )$. In particular, this means that when including common interactions, the functional coefficients in the NMR model (model 1c) are forced to be 0, but this is not so in the URM model (models 3.1c and 3.3c).

To determine consistency, the model fit of the NMR model (model 1[a, b, or c]) and the fit of the URM models (models 3.1(a, b, or c), 3.2(a, b, or c), and 3.3[(a, b, or c]) can be compared; when an URM model is an improved fit, inconsistency may be present. Also, differences between the relative treatment effects and regression coefficients produced from the NMR model and those from the URM models may suggest inconsistency.

### 2.4 Including multi‐arm trials

The models can be applied to data sets including multi‐arm trials providing that the correlation between the observed relative treatment effect (*y*_*ik*_) and the trial‐specific relative treatment effects (*δ*_*i*,1*k*_) is taken into account. For each multi‐arm trial *i* with *m* arms, the observed relative treatment effects and the trial‐specific relative treatment effects are assumed to follow multivariate normal distributions

$$\left(\begin{array}{c} y_{i2}\;\\ \vdots \\ y_{\mathrm{im}}\; \end{array}\right)\sim N\left(\left(\begin{array}{c} \theta_{i2}\\ \vdots \\ \theta_{\mathrm{im}} \end{array}\right),\left(\begin{array}{ccc} v_{i2} & \ldots & \mathrm{cov}\left(y_{i2},y_{\mathrm{im}}\right)\\ \vdots & \ddots & \vdots \\ \mathrm{cov}\left(y_{i2},y_{\mathrm{im}}\right) & \ldots & v_{\mathrm{im}} \end{array}\right)\right)$$

and

$$\left(\begin{array}{c} \delta_{i,12}\;\\ \vdots \\ \delta_{i,1m}\; \end{array}\right)\sim N\left(\left(\begin{array}{c} d_{1,t_{i2}}-d_{1,t_{i1}}\\ \vdots \\ d_{1,t_{\mathrm{im}}}-d_{1,t_{i1}} \end{array}\right),\left(\begin{array}{ccc} \tau^{2} & \ldots & \tau^{2}/2\\ \vdots & \ddots & \vdots \\ \tau^{2}/2 & \ldots & \tau^{2} \end{array}\right)\right).$$

Furthermore, there is an extra consideration when fitting node‐splitting models.^5^, ^6^ If one wants to split node (*t*_*i*1_, *t*_*ik*_), then a multi‐arm trial will contribute direct evidence to the relative treatment effect (*d*^*dir*^) as required because $\hat{t}=t_{i1}$. However, the multi‐arm trial would not contribute direct evidence to the estimation of the relative treatment effect, *d*^*dir*^, if one splits another node (eg,  *t*_*i*2_, *t*_*i*3_) because $\hat{t}\neq t_{i1}$. Therefore, to overcome this problem, when a multi‐arm trial compared the two treatments, t^*^ and $\hat{t}$, in addition to other treatments, treatment $\hat{t}$ is taken to be the baseline treatment *t*_*i*1_ for that study.

Note that for URM models including multi‐arm trial data, the URM model is not the same as fitting separate pair‐wise meta‐regressions because the correlation in multi‐arm trials is taken into account but would not be in pair‐wise analyses; also, the URM model only uses *t*_*i*1_ as the baseline treatment so direct evidence for some pairwise comparisons would not be used whereas pairwise meta‐regression could utilize all direct evidence.

## 3 APPLICATION TO DATA SETS

### 3.1 Data sets

Here, the methods proposed in Section 2 are applied to a real data set and four fabricated data sets that have been manipulated to demonstrate specific scenarios.

#### 3.1.1 Malaria data set

**Figure 2:**
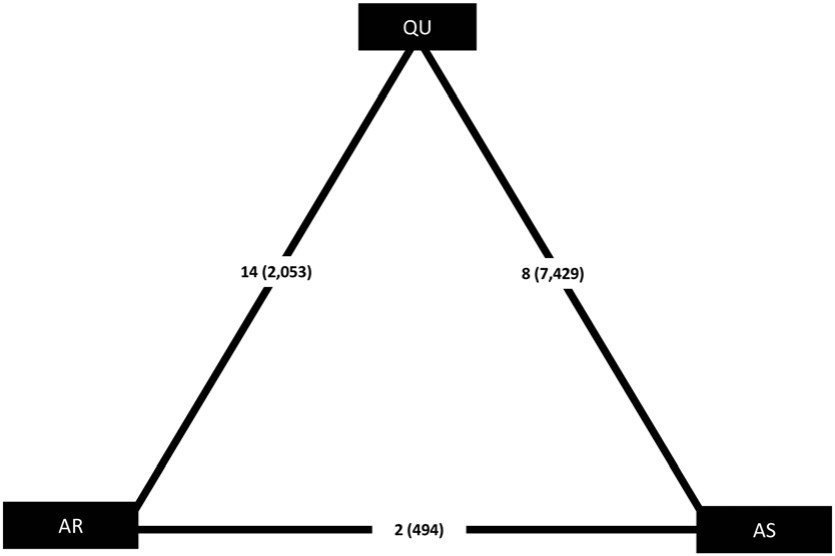
Network diagram for the malaria data set. Number of trials (number of patients) displayed. AR, artemether; AS, artesunate; QU, quinine

Two Cochrane reviews and the corresponding trials were used to construct the malaria data set; reviews compared artemether (AR), quinine (QU), and artesunate (AS).^27^, ^28^ Randomised controlled trials including patients with severe malaria were eligible. Age was considered to be an effect modifier because the clinical features of malaria differ by age and thus, all treatment recommendations are stratified by age in the reviews and World Health Organization treatment guidelines.^29^ Event rates for the primary outcome, death, and the covariate, average age of patients in each trial were extracted. Two studies with missing covariate data were deleted from the data set. Using the event rates, trial‐specific log odds ratios and their standard deviations were calculated in *R*. Table S1 displays the data. Figure 2 shows the network diagram.

#### 3.1.2 Fabricated data sets

Four fabricated data sets were constructed by manipulating the malaria data set to illustrate key scenarios: (a) no interaction is present and the relative treatment effects and regression coefficients are consistent (Figure 1A); (b) interaction exists and the relative treatment effects and coefficients are consistent (Figure 1B); (c) interaction exists, and the relative treatment effects are consistent, but the coefficients are inconsistent (Figure 1D); (4) no interaction is present, and the relative treatment effects are consistent, but the coefficients are inconsistent (Figure 1G). Example R code to generate the data sets is given in the supplementary material.

Analogous to the malaria data set, each data set compared three treatments (AS, AR, QU): there was direct evidence for each possible comparison; no multi‐arm trials contributed; and a dichotomous outcome and continuous covariate was of interest. Ten trials contributed direct evidence to each comparison. For each study, a continuous covariate was taken to be a realization from normal distribution (ie, *N*(17, 10^2^)) truncated at 0 to ensure the covariate values were similar to those observed in the malaria data set.

The log odds ratios and regression coefficients were chosen to be similar to those estimated in the original data set. For each data set, the log odd ratio at 0 covariate of trials comparing treatments AR and AS was 0.2, trials comparing treatments QU and AS was 0.23, and trials of treatments QU and AR was 0.03. For data set one, the coefficient for each comparison was 0. For data set two, the coefficient for trials comparing treatments AR and AS was 0.02, trials comparing treatments QU and AS was 0.02, and trials of treatments QU and AR was 0. For data set three, the coefficient for trials comparing treatments AR and AS was 0.01, trials of treatments QU and AS was 0.04, and trials comparing treatments QU and AR was 0. For data set four, the coefficient for trials comparing treatments AR and AS was −0.04, trials of treatments QU and AS was 0.04, and trials of treatments QU and AR was 0.

The trial‐specific observed log odds ratios were estimated from the values of log odds ratio at 0 covariate, the coefficients, and the covariates. The between‐trial variance was 0. The standard error of the observed log odds ratio was 0.2 for each trial.

### 3.2 Implementation

All models were fitted to the data sets using WinBUGS 1.4.3 and the R2WinBUGS package in R. Example code is provided as supplementary material. For the malaria data set, all models in Table 1 were fitted. For the fabricated data sets, only fixed‐effect versions of models 1a*,* 2.1a, 3.1a, and 4.1a were applied because the between trial variance was 0 and the coefficients differed across comparisons. See Table S2 for the parameterization of the DBT models. The covariates were centered at their mean. All parameters were given noninformative normal prior distributions (ie, *N*(0, 100000)) except the between‐trial standard deviation that was assumed to follow a noninformative uniform distribution (ie, *Uni*(0, 10)) and a weakly informative prior distribution (ie, *uniform* (0, 2)) was specified for the standard deviation of the exchangeable regression coefficients. Three chains with different initial values were run for 300 000 iterations. The initial 100 000 draws were discarded and chains were thinned such that every fifth iteration was retained. Convergence of the chains was assessed by inspecting trace plots of the draws.

Model fit and complexity of models was assessed using the deviance information criterion (DIC) defined as $\mathrm{DIC}=\bar{D}+p_{D}$ where $\bar{D}$ is the posterior mean of the residual deviance and *p*_*D*_ is the effective number of parameters.^30^ A model with a smaller DIC was preferable to a model with a larger DIC, but differences of less than three units were not considered meaningful. When models had little difference in DIC, the simplest model was chosen.

### 3.3 Results

Results from NMR, node‐splitting and URM models are presented here. The results from DBT models are presented in supplementary material.

#### 3.3.1 Malaria data set

##### NMR models

Comparing fixed‐effect and random‐effect NMR models (models 1a, 1b, 1c), the DICs from all NMR models variations are similar (DICs 24.93‐26.76 in Table S3). Also, the estimated regression coefficients for the treatment by average age interactions were quite similar for each model variation (Table S4). Therefore, results from the simplest model to the fixed‐effect NMR with common interactions (model 1c) are presented.

The results of model 1c show that there is evidence of a small interaction between relative treatment effect and average age for AR vs AS and QU vs AS; the posterior median of the common regression coefficient for AR vs AS and QU vs. AS is 0.0132 with 95% credibility interval (CrI), 0.0018‐0.0244, (Table S4). There is no interaction for QU vs AR because the model fixes the coefficient to be 0. However, before using these results to draw clinical inferences, the underlying consistency assumptions must be assessed.

##### Node‐splitting models

Table 2 shows model fit assessment results for fixed‐effect node‐splitting models with common interactions (models 2.1c, 2.2c, 2.3c). The DIC of the NMR model (DIC = 25.29) is similar to those of the node‐splitting models (DICs 23.75‐27.95) indicating that the model is not improved by splitting each node, lending support to the consistency assumptions.

**Table 2:** Model fit assessment results for fixed‐effect models with common treatment by average age interactions for the malaria data set

**Model**	**Mean Residual Deviance**	**p**_**D**_	**DIC**
**NMR model (model 1c)**	22.29	3.00	25.29
**Node‐splitting model splitting the log odds ratio and regression coefficient: AR vs AS (model 2.1c)**	22.97	4.99	27.95
**Node‐splitting model splitting the log odds ratio and regression coefficient: QU vs AS (model 2.1c)**	22.96	4.98	27.93
**Node‐splitting model splitting the log odds ratio and regression coefficient: QU vs AR (model 2.1c)**	20.65	5.00	25.65
**Node‐splitting model splitting the log odds ratio only: AR vs AS (model 2.2c)**	23.27	4.01	27.27
**Node‐splitting model splitting the log odds ratio only: QU vs AS (model 2.2c)**	23.27	4.01	27.29
**Node‐splitting model splitting the log odds ratio only: QU vs AR (model 2.2c)**	23.27	4.01	27.27
**Node‐splitting model splitting the regression coefficient only: AR vs AS (model 2.3c)**	23.19	4.01	27.19
**Node‐splitting model splitting the regression coefficient only: QU vs AS (model 2.3c)**	23.19	4.01	27.19
**Node‐splitting model splitting the regression coefficient only: QU vs AR (model 2.3c)**	19.74	4.01	23.75
**URM model assessing consistency of the log odds ratio and regression coefficient (model 3.1c)**	19.93	4.01	23.94
**URM model assessing consistency of the log odds ratio only (model 3.2c)**	23.27	4.01	27.27
**URM model assessing consistency of the regression coefficient only (model 3.3c)**	18.96	3.00	21.96

Abbreviations: AR, artemether; AS, artesunate; DIC, deviance information criterion; QU, quinine; NMR, network meta‐regression; URM, unrelated mean effects. Number of data points: 24

**Figure 3:**
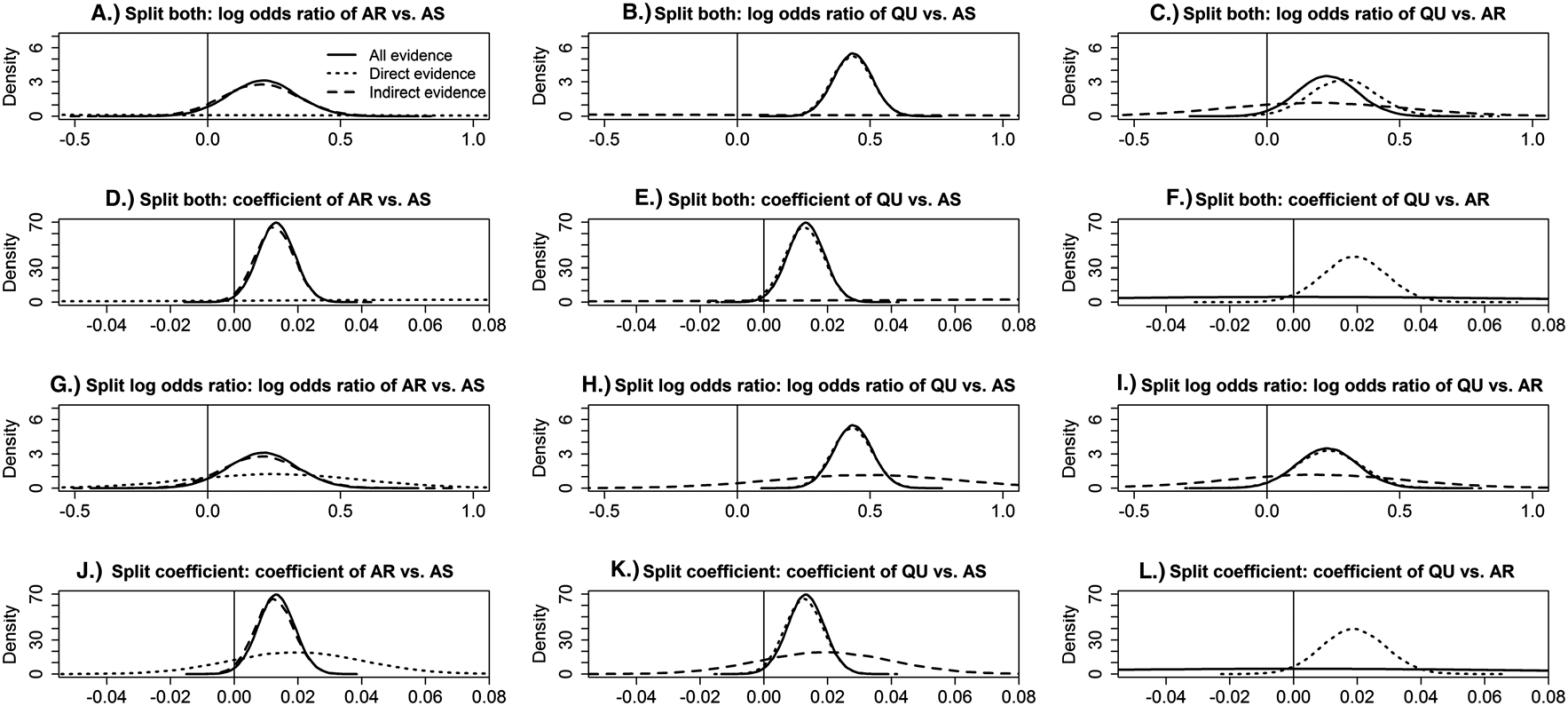
Posterior distributions for the log odds ratios (centered) and regression coefficients for the interaction from fixed‐effect node‐splitting models with common treatment by average age interactions for the malaria data set. Results in Figure A‐F are from models 2.1c and 1c. Results in Figures G‐I are from models 2.2c and 1c. Results in Figures J‐L are from models 2.3c and 1c. In Figures F and I, the coefficient from indirect evidence and from all evidence is forced to be 0. AR, artemether; AS, artesunate; QU, quinine

**Figure 4:**
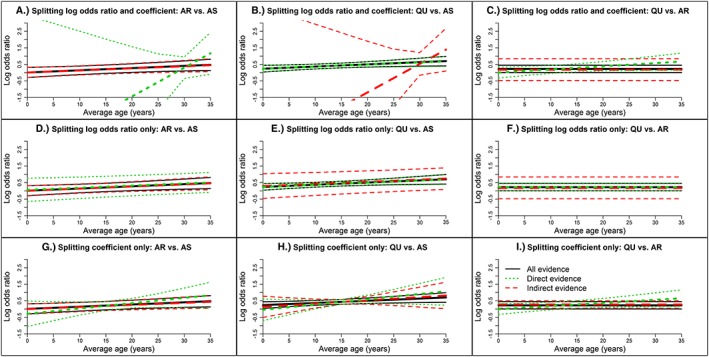
Log odds ratio versus average age for direct and indirect from fixed‐effect node‐splitting models and for all evidence from the fixed‐effect network meta‐regression model with common treatment by average age interactions for the malaria data set. Results in Figures A‐C are from models 2.1c and 1c. Results in Figures D‐F are from models 2.2c and 1c. Results in Figures G‐I are from models 2.3c and 1c. AR, artemether; AS, artesunate; QU, quinine [Colour figure can be viewed at wileyonlinelibrary.com]

The results from node splitting are displayed in Table 3. In the model that assesses consistency of both the log odds ratio and the coefficient (model 2.1c), the log odds ratios for AR vs AS (−2.3540, 95% CrI, −6.7650 to 2.0530) and QU vs AS (0.4316, 95% CrI, 0.2833‐0.5797) based on direct evidence differs with those from indirect evidence (ie, 0.1985, 95% CrI, −0.0815 to 0.4782, and −2.1000, 95% CrI, −6.4180 to 2.4430, respectively) because only two trials contribute direct evidence for AR vs.AS and, therefore, the results are influenced by the vague prior distribution. A similar but less pronounced inconsistency is also seen for the corresponding coefficients. Yet the prob of agreement between direct and indirect evidence is low for the coefficient for QU vs AR (*P* = 0.06) but not remarkably low for other comparisons or the log odds ratios (Ps 0.24‐0.77). Similar conclusions are drawn from models that split either the log odds ratio or the regression coefficient only (models 2.2c and 2.3c). The consistency of the direct and indirect evidence is also supported graphically in Figure 3, which displays the posterior distributions of the centered log odds ratios and regression coefficients and in Figure 4, where the log odds ratio versus average age is plotted.

**Table 3:** Results from fixed‐effect node‐splitting models including common treatment by average age interactions for the malaria data set

**Model Type**	**Parameter**	**Evidence**	**Posterior Median (95% Credibility Interval), *P***		
			**AR vs AS**	**QU vs AS**	**QU vs AR**
**Splitting the log odds ratio and regression coefficient (model 2.1c)**	**Log odds ratio (centered)**	**Direct**	−2.3540 (−6.7650 to 2.0530)*	0.4316 (0.2833‐0.5797)	0.2882 (0.0449‐0.5315)
		**Indirect**	0.1985 (−0.0815 to 0.4782)	−2.1000 (−6.4180 to 2.4430)*	0.1825 (−0.4751 to 0.8419)
		**IE, *P***	−2.5510 (−6.9740 to 1.8710), *P* = 0.26	2.5330 (−2.0150 to 6.8540), *P* = 0.26	0.1055 (−0.5990 to 0.8089), *P* = 0.77
	**Regression coefficient for the interaction**	**Direct**	0.1738 (−0.0974 to 0.4451)	0.0126 (0.0006‐0.0245)	0.0191 (−0.0008 to 0.0387)
		**Indirect**	0.0126 (0.0007‐0.0245)	0.1728 (−0.1048 to 0.4376)	Fixed at 0
		**IE, *P***	0.1613 (−0.1100 to 0.4327), *P* = 0.25	−0.1603 (−0.4253 to 0.1173), *P* = 0.24	0.0191 (−0.0008 to 0.0387), *P* = 0.06
**Splitting the log odds ratio only (model 2.2c)**	**Log odds ratio (centered)**	**Direct**	0.2495 (−0.3804 to 0.8815)	0.4320 (0.2837‐0.5804)	0.2328 (−0.0031 to 0.4700)
		**Indirect**	0.1994 (−0.0821 to 0.4787)	0.4824 (−0.1946 to 1.1600)	0.1816 (−0.4797 to 0.8403)
		**IE, *P***	0.0512 (−0.6481 to 0.7515), *P* = 0.89	−0.0499 (−0.7523 to 0.6552), *P* = 0.89	0.0521 (−0.6518 to 0.7545), *P* = 0.89
	**Regression coefficient for the interaction**	**All**	0.0129 (0.0011‐0.0248)	0.0129 (0.0011‐0.0248)	Fixed at 0
**Splitting the regression coefficient only (model 2.3c)**	**Log odds ratio (centered)**	**All**	0.1890 (−0.0918 to 0.4673)	0.4283 (0.2793‐0.5747)	0.2746 (0.0469‐0.5033)
	**Regression coefficient for the interaction**	**Direct**	0.0195 (−0.0210 to 0.0603)	0.0126 (0.0007‐0.0245)	0.0188 (−0.0007 to 0.0385)
		**Indirect**	0.0125 (0.0007‐0.0245)	0.0194 (−0.0210 to 0.0601)	Fixed at 0
		**IE, *P***	0.0070 (−0.0358 to 0.0500), *P* = 0.75	−0.0068 (−0.0498 to 0.0357), *P* = 0.76	0.0188 (−0.0007 to 0.0385), *P* = 0.06

Abbreviations: AR, artemether; AS, artesunate; IE, inconsistency estimate; *P*, probability of agreement between direct and indirect evidence; QU, quinine.

* Results are influenced by the vague prior distribution and can be considered to be “not estimable.”

##### URM models

Table 2 also displays model fit assessment results for fixed‐effect URM models with common interactions (models 3.1c, 3.2c, 3.3c). The DIC of the NMR model (DIC = 25.29) is similar to those from the URM models the assess consistency of both the log odds ratio and coefficient (DIC = 23.94) or the log odds ratio alone (DIC = 27.27) (models 3.1c and 3.2c) but is slightly higher than that from the model that assesses the coefficient alone (DIC = 21.96) (model 3.3c) indicating a possible inconsistency on a coefficient.

See Table 4 for the results from the NMR model and URM models. The results from the URM models are quite similar to those from the NMR model with the exception of the regression coefficient for QU vs AR. This difference in the coefficient for QU vs AR is because of the different assumptions underlying the two models; the NMR model sets the regression coefficients for AR vs AS and QU vs AS to be identical (ie, 0.0132, 95% CrI, 0.0018‐0.0244) and the coefficient for QU vs AR to be 0, whereas all three coefficients are set to be identical in the URM model (ie, 0.0145, 95% CrI, 0.0044‐0.0247).

**Table 4:** Results from fixed‐effect network meta‐regression and unrelated mean effects models with common treatment by average age interactions for the malaria data set

**Model**	**Parameter**	**Posterior median (95% credibility interval)**		
		**AR vs AS**	**QU vs AS**	**QU vs AR**
**NMR model (model 1c)**	**Log odds ratio (centered)**	0.2080 (−0.0441 to 0.4592)	0.4350 (0.2923‐0.5772)	0.2268 (0.0051‐0.4516)
	**Regression coefficient for the interaction**	0.0132 (0.0018‐0.0244)	0.0132 (0.0018‐0.0244)	Fixed at 0
**URM model assessing consistency of the log odds ratio and regression coefficient (model 3.1c)**	**Log odds ratio (centered)**	0.2229 (−0.4006 to 0.8471)	0.4365 (0.2891‐0.5832)	0.2743 (0.0363‐0.5136)
	**Regression coefficient for the interaction**	0.0145 (0.0044‐0.0247)	0.0145 (0.0044‐0.0247)	0.0145 (0.0044‐0.0247)
**URM model assessing consistency of the log odds ratio only (model 3.2c)**	**Log odds ratio (centered)**	0.2497 (−0.3819 to 0.8806)	0.4317 (0.2831‐0.5794)	0.2328 (−0.0031 to 0.4700)
	**Regression coefficient for the interaction**	0.0128 (0.0011‐0.0248)	0.0128 (0.0011‐0.0248)	Fixed at 0
**URM model assessing consistency of the regression coefficient only (model 3.3c)**	**Log odds ratio (centered)**	0.1725 (−0.0811 to 0.4257)	0.4402 (0.2978‐0.5822)	0.2676 (0.0416‐0.4959)
	**Regression coefficient for the interaction**	0.0148 (0.0048‐0.0246)	0.0148 (0.0048‐0.0246)	0.0148 (0.0048‐0.0246)

Abbreviations: AR, artemether; AS, artesunate; NMR, network meta‐regression; QU, quinine; URM, unrelated mean effects.

Overall, there is not only evidence of an interaction from the NMR but also evidence of inconsistency; the node‐splitting models show evidence of loop inconsistency for the coefficient of QU vs AR, and the URM models support this showing a possible inconsistency of the coefficients.

#### 3.3.2 Fabricated data sets

##### Data set 1: No interaction and consistency

**Figure 5:**
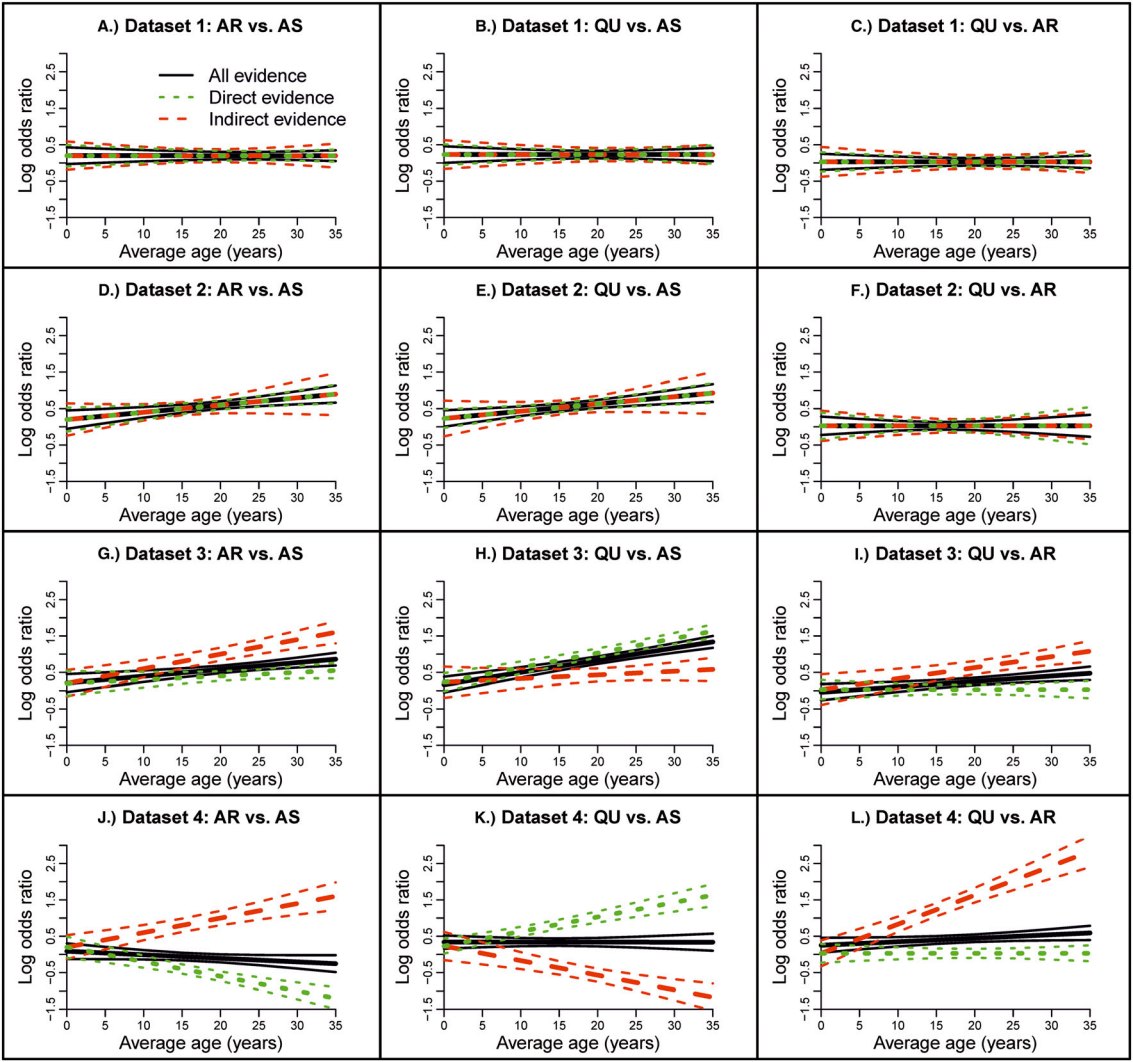
Log odds ratio versus average age for direct and indirect from fixed‐effect node‐splitting models (model 2.1a) and for all evidence from the fixed‐effect network meta‐regression model (model 1a) with independent treatment by average age interactions for the fabricated data sets. AR, artemether; AS, artesunate; QU, quinine [Colour figure can be viewed at wileyonlinelibrary.com]

The DICs from each model (models 1a, 2.1a, and 3.1a) are similar (8.01‐12.00); therefore, there is no obvious sign of inconsistency (Table 5). Using the results from node splitting (model 2.1a), the log odds ratios and coefficients based on direct and indirect evidence are very similar, and the probabilities of agreement between direct and indirect evidence are practically one (Table 6). The results from the NMR model are also similar to those from the URM model (model 3.1a) (Table 7) indicating consistency. Overall, the NMR model does not show that a treatment by average age interaction exists (Table 7) and there is no evidence of loop inconsistency using node splitting or global inconsistency using the URM model. Figure 5, which shows the results from the NMR model and node‐splitting models, supports this conclusion.

**Table 5:** Model fit assessment results for fixed‐effect models assessing consistency of both the log odds ratio and regression coefficient with independent treatment by average age interactions for the fabricated data sets

**Data Set**	**Model**	**Mean Residual Deviance**	**p**_**D**_	**DIC**
**Data set 1: No interaction and consistency**	**NMR model (model 1a)**	4.00	4.00	8.01
	**Node‐splitting model: AR vs AS (model 2.1a)**	6.00	6.00	12.00
	**Node‐splitting model: QU vs AS (model 2.1a)**	5.99	5.99	11.98
	**Node‐splitting model: QU vs AR (model 2.1a)**	5.99	5.99	11.98
	**URM model (model 3.1a)**	5.99	5.99	11.97
**Data set 2: Interaction and consistency**	**NMR model (model 1a)**	4.00	4.00	8.00
	**Node‐splitting model: AR vs AS (model 2.1a)**	6.00	6.00	11.99
	**Node‐splitting model: QU vs AS (model 2.1a)**	5.99	5.99	11.99
	**Node‐splitting model: QU vs AR (model 2.1a)**	5.99	5.99	11.97
	**URM model (model 3.1a)**	5.98	5.98	11.97
**Data set 3: Interaction and inconsistency**	**NMR model (model 1a)**	43.14	3.99	47.14
	**Node‐splitting model: AR vs AS (model 2.1a)**	5.99	5.99	11.99
	**Node‐splitting model: QU vs AS (model 2.1a)**	6.00	6.00	11.99
	**Node‐splitting model: QU vs AR (model 2.1a)**	5.98	5.98	11.97
	**URM model (model 3.1a)**	5.99	5.99	11.97
**Data set 4: No interaction and inconsistency**	**NMR model (model 1a)**	184.36	4.00	188.36
	**Node‐splitting model: AR vs AS (model 2.1a)**	6.00	6.00	12.00
	**Node‐splitting model: QU vs AS (model 2.1a)**	5.99	5.99	11.99
	**Node‐splitting model: QU vs AR (model 2.1a)**	6.00	6.00	11.99
	**URM model (model 3.1a)**	5.99	5.99	11.98

Abbreviations: AR, artemether; AS, artesunate; DIC, deviance information criterion; QU, quinine; NMR, network meta‐regression; URM, unrelated mean effects. Number of data points: 30.

**Table 6:** Results from fixed‐effect node‐splitting models splitting both the log odds ratio and regression coefficient including independent treatment by average age interactions (model 2.1a) for the fabricated data sets

**Data Set**	**Parameter**	**Evidence**	**Posterior Median (95% Credibility Interval), *P***		
			**AR vs AS**	**QU vs AS**	**QU vs AR**
**Data set 1: No interaction and consistency**	**Log odds ratio (uncentered)**	**Direct**	0.1997 (−0.0948 to 0.4949)	0.2302 (−0.0566 to 0.5139)	0.0298 (−0.2356 to 0.2937)
		**Indirect**	0.2001 (−0.1865 to 0.5902)	0.2306 (−0.1642 to 0.6265)	0.0297 (−0.3799 to 0.4398)
		**IE, *P***	−0.0007 (−0.4870 to 0.4894), *P* = 0.9974	−0.0004 (−0.4879 to 0.4875), *P* = 0.9986	−0.0002 (−0.4891 to 0.4886), *P* = 0.9990
	**Regression coefficient for the interaction**	**Direct**	0.0000 (−0.0107 to 0.0109)	0.0000 (−0.0135 to 0.0136)	0.0000 (−0.0115 to 0.0116)
		**Indirect**	0.0000 (−0.0178 to 0.0178)	0.0000 (−0.0158 to 0.0158)	0.0000 (−0.0174 to 0.0174)
		**IE, *P***	0.0000 (−0.0210 to 0.0208), *P* = 0.9980	0.0000 (−0.0208 to 0.0209), *P* = 0.9980	0.0000 (−0.0208 to 0.0209), *P* = 0.9982
**Data set 2: Interaction and consistency**	**Log odds ratio (uncentered)**	**Direct**	0.1992 (−0.1284 to 0.5285)	0.2300 (−0.0268 to 0.4852)	0.0301 (−0.3372 to 0.3941)
		**Indirect**	0.1998 (−0.2432 to 0.6460)	0.2304 (−0.2614 to 0.7213)	0.0299 (−0.3886 to 0.4447)
		**IE, *P***	−0.0007 (−0.5528 to 0.5534), *P* = 0.9980	−0.0001 (−0.5549 to 0.5537), *P* = 0.9998	−0.0003 (−0.5542 to 0.5548), *P* = 0.9996
	**Regression coefficient for the interaction**	**Direct**	0.0200 (0.0049‐0.0352)	0.0200 (0.0069‐0.0333)	0.0000 (−0.0239 to 0.0240)
		**Indirect**	0.0200 (−0.0073 to 0.0473)	0.0199 (−0.0084 to 0.0485)	0.0000 (−0.0200 to 0.0201)
		**IE, *P***	0.0000 (−0.0313 to 0.0312), *P* = 0.9974	0.0001 (−0.0315 to 0.0313), *P* = 0.9954	0.0000 (−0.0311 to 0.0313), *P* = 1.0000
**Data set 3: Interaction and inconsistency**	**Log odds ratio (uncentered)**	**Direct**	0.2000 (−0.1389, 0.5372)	0.2301 (−0.0208, 0.4796)	0.0301 (−0.2355 to 0.2937)
		**Indirect**	0.1999 (−0.1619, 0.5649)	0.2304 (−0.1985, 0.6584)	0.0299 (−0.3924 to 0.4492)
		**IE, *P***	0.0003 (−0.4955 to 0.4950), *P* = 0.9990	−0.0006 (−0.4948 to 0.4955), *P* = 0.9982	−0.0004 (−0.4971 to 0.4983), *P* = 0.9986
	**Regression coefficient for the interaction**	**Direct**	0.0100 (−0.0039 to 0.0241)	0.0400 (0.0298‐0.0503)	0.0000 (−0.0125, 0.0126)
		**Indirect**	0.0400 (0.0237‐0.0562)	0.0099 (−0.0088 to 0.0289)	0.0300 (0.0127‐0.0474)
		**IE, *P***	−0.0300 (−0.0515 to −0.0088), *P* = 0.0059	0.0301 (0.0085‐0.0514), *P* = 0.0062	−0.0300 (−0.0515 to −0.0086), *P* = 0.0057
**Data set 4:** **No interaction and inconsistency**	**Log odds ratio (uncentered)**	**Direct**	0.2002 (−0.0926 to 0.4908)	0.2300 (0.0222‐0.4360)	0.0297 (−0.2260 to 0.2863)
		**Indirect**	0.2000 (−0.1290, 0.5298)	0.2300 (−0.1569, 0.6178)	0.0301 (−0.3279 to 0.3866)
		**IE, *P***	−0.0003 (−0.4376 to 0.4397), *P* = 0.9990	−0.0007 (−0.4393 to 0.4399), *P* = 0.9976	0.0000 (−0.4398 to 0.4398), *P* = 1.0000
	**Regression coefficient for the interaction**	**Direct**	−0.0400 (−0.0553 to −0.0246)	0.0400 (0.0273 to 0.0529)	0.0000 (−0.0115 to 0.0116)
		**Indirect**	0.0399 (0.0227, 0.0574)	−0.0400 (−0.0591, −0.0208)	0.0800 (0.0600‐0.1000)
		**IE, *P***	−0.0799 (−0.1031 to −0.0571), *P* = 0.0000	0.0800 (0.0568‐0.1030), *P* = 0.0000	−0.0800 (−0.1031 to −0.0569), *P* = 0.0000

Abbreviations: AR, artemether; AS, artesunate; IE, inconsistency estimate; *P*, probability of agreement between direct and indirect evidence; QU, quinine. Posterior median (95% credibility interval) presented.

**Table 7:** Results from fixed‐effect network meta‐regression and unrelated mean effects models assessing consistency of both the log odds ratio and regression coefficient with independent treatment by average age interactions for the fabricated data sets

**Data Set**	**Model**	**Parameter**	**Posterior Median (95% Credibility Interval)**		
			**AR vs AS**	**QU vs AS**	**QU vs AR**
**Data set 1: No interaction and consistency**	**NMR model (model 1a)**	**Log odds ratio (uncentered)**	0.2002 (−0.0305 to 0.4281)	0.2302 (0.0014‐0.4587)	0.0306 (−0.1911 to 0.2517)
		**Regression coefficient for the interaction**	0.0000 (−0.0090 to 0.0091)	0.0000 (−0.0102 to 0.0102)	0.0000 (−0.0096 to 0.0096)
	**URM model (model 3.1a*)***	**Log odds ratio (uncentered)**	0.2002 (−0.0947 to 0.4926)	0.2301 (−0.0556 to 0.5148)	0.0303 (−0.2340 to 0.2937)
		**Regression coefficient for the interaction**	0.0000 (−0.0108 to 0.0108)	0.0000 (−0.0135 to 0.0136)	0.0000 (−0.0116 to 0.0116)
**Data set 2: Interaction and consistency**	**NMR model (model 1a)**	**Log odds ratio (uncentered)**	0.2006 (−0.0539 to 0.4514)	0.2302 (0.0043‐0.4558)	0.0298 (−0.2223 to 0.2828)
		**Regression coefficient for the interaction**	0.0200 (0.0074‐0.0327)	0.0200 (0.0080‐0.0321)	0.0000 (−0.0147 to 0.0147)
	**URM model (model 3.1a*)***	**Log odds ratio (uncentered)**	0.2000 (−0.1289 to 0.5266)	0.2301 (−0.0264 to 0.4856)	0.0302 (−0.3364 to 0.3948)
		**Regression coefficient for the interaction**	0.0200 (0.0049‐0.0351)	0.0200 (0.0068‐0.0332)	0.0000 (−0.0240 to 0.0240)
**Data set 3: Interaction and inconsistency**	**NMR model (model 1a)**	**Log odds ratio (uncentered)**	0.2081 (−0.0390 to 0.4523)	0.1654 (−0.0503 to 0.3808)	−0.0421 (−0.2636 to 0.1801)
		**Regression coefficient for the interaction**	0.0187 (0.0082‐0.0292)	0.0335 (0.0244‐0.0425)	0.0147 (0.0047‐0.0248)
	**URM model (model 3.1a*)***	**Log odds ratio (uncentered)**	0.2003 (−0.1374 to 0.5353)	0.2301 (−0.0201 to 0.4795)	0.0303 (−0.2340 to 0.2938)
		**Regression coefficient for the interaction**	0.0100 (−0.0040 to 0.0240)	0.0400 (0.0297‐0.0503)	0.0000 (−0.0125 to 0.0125)
**Data set 4: No interaction and inconsistency**	**NMR model (model 1a)**	**Log odds ratio (uncentered)**	0.0877 (−0.1296 to 0.3034)	0.3389 (0.1566‐0.5214)	0.2515 (0.0472‐0.4567)
		**Regression coefficient for the interaction**	−0.0098 (−0.0211 to 0.0017)	−0.0001 (−0.0105 to 0.0103)	0.0097 (−0.0002 to 0.0195)
	**URM model (model 3.1a*)***	**Log odds ratio (uncentered)**	0.2004 (−0.0911 to 0.4899)	0.2302 (0.0231‐0.4372)	0.0305 (−0.2259 to 0.2854)
		**Regression coefficient for the interaction**	−0.0400 (−0.0553 to −0.0247)	0.0400 (0.0272‐0.0529)	0.0000 (−0.0115 to 0.0116)

Abbreviation: AR, artemether; AS, artesunate; NMR, network meta‐regression; QU, quinine; URM, unrelated mean effects.

##### Data set 2: Interaction and consistency

The DICs from the models (models 1a, 2.1a, and 3.1a) are again similar (8.00‐11.99) indicating consistent evidence (Table 5). From node splitting (model 2.1a), the log odds ratios and the coefficients based on direct and indirect evidence are almost identical, and the probabilities of agreement of direct and indirect evidence are practically one (Table 6); Figure 5 shows the results graphically. The URM model (model 3.1a) also gives comparable results to the NMR model (Table 7). In conclusion, the NMR model shows that an interaction exists for AR vs AS (0.0200, 95% CrI, 0.0074‐0.0327) and QU vs AS (0.0200, 95% CrI, 0.0080‐0.0321) (Table 7) and there is no loop inconsistency using node splitting, or global inconsistency using the URM model.

##### Data set 3: Interaction and inconsistency

The DIC from the NMR model (model 1a) (DIC = 47.14) is much higher than those from node splitting (model 2.1a) and the URM model (model 3.1a) (11.97‐11.99) suggesting inconsistency (Table 5). From node splitting, the log odds ratios based on direct and indirect evidence are comparable, but the coefficients for AR vs AS (0.0100, 95% CrI, −0.0039 to 0.0241) and QU vs AS (0.0400, 95% CrI, 0.0298 to 0.0503) and QU vs AR (0.0000, 95% CrI, −0.0125 to 0.0126) from direct evidence differ from those from indirect evidence (ie, 0.0400, 95% CrI, 0.0237‐0.0562, 0.0099, 95% CrI, −0.0088 to 0.0289, and 0.0300, 95% CrI, 0.0127‐0.0474, respectively); the probabilities of agreement of direct and indirect evidence are very high (Ps 0.9982‐0.9990) for the log odds ratios and very low for the coefficients (Ps 0.0057‐0.0062) (Table 6). The URM model also gives results that differ somewhat from those of the NMR model (see Table 7). To summarise, the NMR model shows that an interaction exists for AR vs AS (0.0187, 95% CrI, 0.0082‐0.0292), QU vs AS (0.0335, 95% CrI, 0.0244‐0.0425), and QU vs AR (0.0147, 95% CrI, 0.0047‐0.0248) (Table 7) but there is also loop inconsistency in the size of the underlying coefficients based on direct and indirect evidence that is seen using node splitting (Figure 5); the URM model identifies global inconsistency.

##### Data set 4: No interaction and inconsistency

The DIC from the NMR model (model 1a) (DIC = 188.36) is much higher than those from node splitting (model 2.1a) and the URM model (model 3.1a) (11.99‐12.00) indicating inconsistency (Table 5). Similar to data set 3, in node‐splitting models, the log odds ratios based on direct and indirect evidence are comparable, but the coefficients for AR vs. AS (−0.0400, 95% CrI, −0.0553 to −0.0246) and QU vs. AS (0.0400, 95% CrI, 0.0273‐0.0529) and QU vs AR (0.0000, 95% CrI, −0.0115 to 0.0116) from direct evidence differ from those from indirect evidence (ie, 0.0399, 95% CrI, 0.0227‐0.0574, −0.0400, 95% CrI, −0.0591 to −0.0208, and 0.0800, 95% CrI, 0.0600‐0.1000, respectively); the probabilities of agreement of direct and indirect evidence are very high for log odds ratios (Ps 0.9976‐1.000) and 0 for the coefficients (Table 6). Also, results from the URM model are different from those of the NMR model (see Table 7). Overall, the NMR model shows that no interaction exists (Table 7) but there is inconsistency in the direction of the underlying coefficients based on direct and indirect evidence and this trend can be seen using node splitting (Figure 5); the URM model suggests global inconsistency respectively, but these models cannot show the underlying trend.

## 4 DISCUSSION

We have shown that node‐splitting and inconsistency models can be useful for assessing the underlying consistency assumptions of NMR when using aggregate data. Once consistency has been assessed, the analyst must decide which results to present. If the direct and indirect evidence are consistent, the results from the NMR should be reliable. However, the level of heterogeneity (from the NMR or standard pairwise analyses) and goodness of fit of the NMR should be considered when drawing conclusions from the results. If there is inconsistency, the results from the NMR are questionable and the causes of inconsistency should be considered. In some scenarios, for example, when inconsistency masks an interaction, as shown in Figure 1C,G, the results would not be useable. If the original purpose of the NMR was to explore causes of heterogeneity or inconsistency in an NMA and there is no interaction and no inconsistency masking interactions in the NMR, then analysts could proceed by exploring other potentially relative treatment effect modifying covariates or reconsidering the eligibility criteria.

Each of the proposed methods has different pros and cons. DBT models assess design and loop consistency and can assess global inconsistency, while node splitting assesses loop consistency and URM models assess global inconsistency; loop inconsistency is well recognized in the methodological literature but design consistency is a newer concept.^7^, ^11^ Furthermore, the DBT model requires parameterization by the analyst; therefore, the analyst needs to have a good understanding of the model and parameters. Key advantages of the DBT model and node splitting is that IEs and the prob that direct and indirect evidence agree can be obtained; however, the URM model does not provide such results. Moreover, concerns regarding multiple testing may apply to node‐splitting and the DBT models where probabilities are calculated, particularly when a Frequentist approach is taken; therefore, it is important to compare model fit statistics across models, and also to be cautious in interpreting “*P* values” making sure to allow for multiple testing. One disadvantage of node splitting is that, as one model is fitted for every comparison with contributing direct and indirect evidence, many models may need to be fitted, which is computationally demanding, whereas only one inconsistency model would need to be applied.

Ideally, all three approaches (ie, node‐splitting model, DBT model, and URM model) would be applied to provide a thorough assessment of consistency. However, in practice, the reviewer may select their preferred approach depending on the ease of application in software etc. We recommend that at least one of the global tests (ie, inconsistency models) and also node splitting are performed. Our preference is node splitting because estimates from direct and indirect evidence can be found.

We proposed and applied methods to trial‐level aggregated data in this article. However, it is straightforward to adapt the models to accommodate any type of arm‐level outcome data, that is, a summary of the outcome data for each arm of each trial and a covariate value for each trial. To adapt the models, a suitable link function would be chosen and nuisance parameters are included in the model to represent the effect of the baseline treatment in Arm 1 of trial *i*. Further details regarding arm‐level NMA models are given by Dias, Sutton, Ades, and Welton^31^

Moreover, collection and use of individual patient data is generally advantageous over aggregate data when studying patient‐level covariates because they avoid ecological biases.^32^, ^33^ Yet it is more common to explore patient‐level covariates (eg, patient age) using study‐level covariate summaries (eg, average age of patients) in meta‐regression such as in the malaria data set. However, when using aggregate data, the possibility of confounding and ecological biases should be considered when patient‐level covariates are explored.

There are a number of issues that can arise when applying the methods, particularly with aggregate data. Parameter estimation can be a problem with limited data, such that models cannot be fitted at all, interactions exist but cannot be detected, or inconsistency exists but is not found. For instance, when all the trials that contribute to the estimation of a regression coefficient have the same covariate value or when only one trial contributes to a coefficient, this would preclude the use of models with independent interactions, but analysts may be able to apply a model with exchangeable or common interactions providing studies that contribute to another basic coefficient that has different covariate values. For example, when exploring an interaction between relative treatment effect and study location (ie, continent), studies that contribute to results for Comparison 2 vs 1 may all be carried out on the same continent provided that studies that contribute to Comparison 3 vs 1 are located on different continents. Parameter estimation may particularly be a problem when fitting the DBT model because the IEs would be imprecise when the number of trials in one or more designs is limited; to overcome this one could assume exchangeability of the inconsistency factors or use informative prior distributions. Similarly, if direct evidence is limited for some comparisons (ie, few trials or covariate values), the URM model and node‐splitting models would produce imprecise results, and informative prior distributions may need to be used. Ideally, any informative prior distributions would be evidence based by eliciting them from similar meta‐analyses or experts' beliefs. Finally, it is also worth emphasising that no evidence of inconsistency does not automatically imply there is consistency; inconsistency may exist but cannot be detected when data are limited and results are imprecise, and therefore, arguably the consistency assumptions and the NMR results are questionable. In the same way, in such cases, no evidence of a treatment by covariate interaction does not imply there is truly no interaction.

Conversely, with abundant data, additional modelling extensions may be feasible. For example, in node‐splitting models, we have assumed the between‐trial variance is the same for direct evidence and indirect evidence, yet it is possible to incorporate two variances, one of each type of evidence. Also, the models could be adapted to include more than one covariate or other variance structures.^34^

In conclusion, consistency of the assumptions underlying NMR must be assessed when NMR is applied, even when no treatment by covariate interactions are detected. It is possible that inconsistency is masking an interaction. Furthermore, results of an NMR should not be reported without assessing the underlying assumptions to determine whether the results are valid and reliable.

## CONFLICT OF INTEREST

The authors have declared no competing interests exist.

## AUTHOR CONTRIBUTIONS

SDo proposed extending the existing node‐splitting models proposed by SDi and NW and inconsistency models to include treatment by covariate interactions. NW proposed additional modelling extensions. SDo carried out the analysis and wrote the first draft of the manuscript. SDi and NW provided statistical guidance and commented on the manuscript.

## Supporting information

Table S1. Malaria dataset.
Table S2. Parameterisation of the DBT model that assess consistency of both the log odds ratio and the regression coefficient with independent treatment by average age interactions (*model 4.1a*) for the malaria and fabricated datasets.
Table S3. Model fit assessment results and between‐site variances for fixed‐effect and random‐effects NMR models for the malaria dataset.
Table S4. Regression coefficients for the treatment by average age interaction from fixed‐effect and random‐effects NMR models for the malaria dataset.
Table S5. Model fit assessment results for fixed‐effect NMR and DBT models with common treatment by average age interactions for the malaria dataset.
Table S6. Results from fixed‐effect NMR and DBT models with common treatment by average age interactions for the malaria dataset.
Table S7. Model fit assessment results for fixed‐effect NMR and DBT models assessing consistency of both the log odds ratio and regression coefficient with independent treatment by average age interactions for the fabricated datasets.
Table S8. Results from fixed‐effect NMR and DBT models assessing consistency of both the log odds ratio and regression coefficient with independent treatment by average age interactions for the fabricated datasets.
