# Supplementary material for: Assessing the consistency assumptions underlying network meta‐regression using aggregate data
Source: Res Synth Methods. 2018 Nov 12;10(2):207–24. doi: 10.1002/jrsm.1327 (PMC6563470; doi:10.1002/jrsm.1327)
Supplement: Supplementary file 1 — Table S1. Malaria dataset. Table S2. Parameterisation of the DBT model that assess consistency of both the log odds ratio and the regression coefficient with independent treatment by average age interactions (model 4.1a) for the malaria and fabricated datasets. Table S3. Model fit assessment results and between‐site variances for fixed‐effect and random‐effects NMR models for the malaria dataset. Table S4. Regression coefficients for the treatment by average age interaction from fixed‐effect and random‐effects NMR models for the malaria dataset. Table S5. Model fit assessment results for fixed‐effect NMR and DBT models with common treatment by average age interactions for the malaria dataset. Table S6. Results from fixed‐effect NMR and DBT models with common treatment by average age interactions for the malaria dataset. Table S7. Model fit assessment results for fixed‐effect NMR and DBT models assessing consistency of both the log odds ratio and regression coefficient with independent treatment by average age interactions for the fabricated datasets. Table S8. Results from fixed‐effect NMR and DBT models assessing consistency of both the log odds ratio and regression coefficient with independent treatment by average age interactions for the fabricated datasets. [file JRSM-10-207-s001.pdf]

Supplementary material

METHODS FOR ASSESSING CONSISTENCY USING DBT MODELS

DBT models aim to assess and quantify design and loop inconsistency, where design inconsistency is the difference between results from different study designs. For DBT models, study design is defined as the set of treatments allocated in the study. A test for global inconsistency has been proposed elsewhere when fitting DBT models (Higgins et al., 2012; White et al., 2012).

To set notation, let g be the design of the study where $g = (1, \dots, G)$. For instance, consider a network of three treatments (i.e. *1, 2, 3*) with trials of four different designs: design 1= {*1, 2*}, design 2={1, *3*}, design 3={2, *3*} and design 4={*1, 2, 3*}. The parameterisation of the model is decided in advance. One parameterisation would enable trials of design {1, *2*} to estimate the relative treatment effect d_{12} and the regression coefficient β_{12} of 2 vs. *1*; trials of design {1, *3*} to estimate the relative treatment effect d_{13} and coefficient β_{13} of 3 vs. *1*; trials of design {2, *3*} to estimate two relative treatment effects d_{12} and $(d_{13} + \omega_{3,13})$ and two coefficients β_{12} and $(\beta_{13} + \varphi_{3,13})$; and trials of design {*1, 2, 3*} to estimate two relative treatment effects $(d_{12} + \omega_{4,12})$ and $(d_{13} + \omega_{4,13})$ and two coefficients $(\beta_{12} + \varphi_{4,12})$ and $(\beta_{13} + \varphi_{4,13})$. Here, $\omega_{3,13}$ is the loop inconsistency parameter for *3* vs. *2* from trials of design {*2, 3*} compared with indirect evidence from trials of design {*1, 2*} and trials of design {*1, 3*} and $\varphi_{3,13}$ is the corresponding inconsistency parameter for the coefficient. Also, $\omega_{4,12}$ is the design inconsistency parameter for *2* vs. *1* in trials of design {*1, 2, 3*} compared with trials of design {*1, 2*} and $\omega_{4,13}$ is the design inconsistency parameter for *3* vs. *1* in design {*1, 2, 3*} compared with design {*1, 3*}; $\varphi_{4,12}$ and $\varphi_{4,13}$ are the analogous inconsistency parameters for

the coefficients. The subscript of the inconsistency parameter denotes the design of the studies that estimate it and the treatment comparison.

Extending *model 1a,* the DBT model that assesses the consistency of the relative treatment effects and coefficients and includes independent interactions (*model 4.1a*) is

$$\theta_{ik} = \delta_{i,\,1k} + \beta_{t_{i1},t_{ik}} x_i + \omega_{g,1k} + \varphi_{g,1k} x_i$$

where $\omega_{g,1k}$ reflects inconsistency in the relative treatment effect of t_{ik} vs. t_{i1} (with $k \geq 2$) and $\varphi_{g,1k}$ represents inconsistency in the regression coefficient of t_{ik} vs. t_{i1} (with $k \geq 2$). In this article, inconsistency parameters are assumed to be fixed, although random inconsistency effects are possible and described elsewhere (Higgins et al., 2012; Jackson et al., 2014; Law et al., 2016).

The DBT model that assesses only consistency of the relative treatment effects and includes independent interactions (*model 4.2a*) is the same as model *4.1a* but with the term $\varphi_{g,1k} x_i$ omitted. Similarly, the DBT model that assesses consistency of the coefficients alone and includes independent interactions (*model 4.3a*) is given by deleting then term $\omega_{g,1k}$ from model *4.1a.* Exchangeable (*models 4.1b, 4.2b, 4.3b*) or common (*models 4.1c, 4.2c, 4.3c*) interactions are also possible.

To draw conclusions regarding consistency, the model fit of the NMR model (*model 1(a, b, or c)*) can be compared with the fit of the DBT models (*models 4.1(a, b, or c), 4.2(a, b, or c)* and *4.3(a, b, or c)*); inconsistency is indicated when a DBT model is a better fit. Also, the size, direction and precision of the inconsistency parameters can be considered; larger

inconsistency parameters are a sign of inconsistency. White et al compared the size of the inconsistency factor with its standard error which may help assess inconsistency (Higgins et al., 2012; White et al., 2012). Using Bayesian methods, the probability that each inconsistency parameter is greater than or equal to zero (i.e. $prob2$) can also be calculated by counting the number of iterations for which the parameter is greater than or equal to zero and dividing the number of counted iterations by the total number of iterations of the chain. Because inconsistency can be in either direction this is adjusted to give a ‘two-sided probability’ given by $P = 2 \times minimum(prob2, 1 - prob2)$ (Marshall and Spiegelhalter, 2007).

RESULTS FOR ASSESSING CONSISTENCY USING DBT MODELS

Malaria dataset

The model fit assessment results for fixed-effect DBT models with common interactions (*models 4.1c, 4.2c, 4.3c*) are given in Table S5. The DIC of the NMR model (DIC=25.29) and the DBT models (DICs 23.75-27.29) are similar indicating consistency.

Table S6 shows the results from the NMR model and DBT models. The log odds ratios and coefficients from the DBT models are agreeable with those from the NMR model. In the model that assesses consistency of both the log odds ratio and the coefficient (*model 4.1c*), the probability of agreement between direct and indirect evidence for QU vs. AR is low for the coefficient (P=0.06) but not for the log odds ratio (P=0.77). The results of the models that assess consistency of either the log odds ratio or the coefficient (*models 4.2c and 4.3c*) show comparable findings.

Fabricated datasets

Dataset 1: no interaction and consistency.

The DICs from *models 1a* and *4.1a* are similar (8.01-11.98) therefore there is no obvious sign of inconsistency (Table S7). The DBT model (*model 4.1a*) produces log odds ratios and coefficient that are comparable with those of the NMR model (*model 1a*) and the probabilities of agreement are very high (P=0.9986 and P=0.9996) (Table S8) therefore there is no evidence of loop inconsistency.

Dataset 2: interaction and consistency.

The DICs from the models (*models 1a, 4.1a*) are again similar (8.00-11.97) indicating consistent evidence (Table S7). Again, the DBT model (*model 4.1a*) produces similar results to those of the NMR model (*model 1a*) and the probabilities of agreement are high (P=0.9984 and P=0.9970) (Table S8) therefore there is no loop inconsistency.

Dataset 3: interaction and inconsistency.

The DIC from the NMR model (*model 1a*) (DIC=47.14) is much higher than that from the DBT model (*model 4.1a*) (11.98) suggesting inconsistency (Table S7). In the DBT model, the results differ slightly from those of the NMR model and the agreement probabilities are very low (Ps 0.0000-0.0060) indicating loop inconsistency for the log odds ratio and coefficient. (Table S8).

Dataset 4: no interaction and inconsistency.

The DIC from the NMR model (*model 1a*) (DIC=188.36) is much higher than that from the DBT model (*model 4.1a*) (2.00) showing inconsistency (Table S7). The results from the DBT model differ from those of the NMR model and the probabilities of agreement are zero suggesting loop inconsistency (Table S8).

SUPPLEMENTARY TABLES

Trial	Event rates (number of patients with events/total number of patients)						Log odds ratio	Standard deviation	Average age (years)
	Artesunate		Artemether		Quinine				
**Phu 2010**	13	186	24	184	-	-	0.69	0.36	32.25
**Vinh 1997**	10	79	5	45	-	-	-0.15	0.58	27.33
**Anh 1989**	2	19	-	-	7	22	1.38	0.88	34.47
**Anh 1995**	8	99	-	-	18	91	1.03	0.45	30.95
**Cao 1997**	4	37	-	-	5	35	0.32	0.72	5.50
**Dondorp 2005**	107	730	-	-	164	731	0.52	0.14	27.90
**Dondorp 2010**	230	2712	-	-	297	2713	0.28	0.09	2.85
**Eltahir 2010**	1	33	-	-	2	33	0.72	1.25	4.50
**Hien 1992**	5	31	-	-	8	30	0.64	0.64	28.50
**Newton 2003**	7	59	-	-	12	54	0.75	0.52	25.00
**Adam 2002**	-	-	0	20	1	21	1.10	1.66	3.84
**Aguwa 2010**	-	-	7	44	6	46	-0.23	0.60	3.50
**Hien 1996**	-	-	36	284	47	276	0.35	0.24	30.00
**Huda 2003**	-	-	5	23	6	23	0.24	0.69	6.20
**Karbwang 1992**	-	-	1	14	5	12	2.23	1.19	31.05
**Karbwang 1995**	-	-	6	47	19	50	1.43	0.53	26.50
**Minta 2005**	-	-	4	33	2	34	-0.79	0.90	6.80
**Murphy 1996**	-	-	18	89	8	71	-0.69	0.46	2.33
**Ojuawo 1998**	-	-	1	18	2	19	0.69	1.27	3.93
**Olumese 1999**	-	-	11	54	14	49	0.45	0.46	3.15
**Osonuga 2009**	-	-	0	16	0	16	0.00	2.03	7.00
**Taylor 1998**	-	-	9	83	13	81	0.45	0.47	3.08
**van Hensbroek 1996**	-	-	59	288	62	288	0.06	0.20	3.92
**Walker 1993**	-	-	3	25	6	29	0.65	0.77	3.00

Table S1: Malaria dataset.
Drugs were coded as artesunate=1, artemether=2, and quinine=3.

Design group	Treatments	Treatment 1	Treatment 2	Treatment 3
1	$\{1, 2\}$	Reference	$d_{12} + \beta_{12}x_i$	-
2	$\{1, 3\}$	Reference	-	$d_{13} + \beta_{13}x_i$
3	$\{2, 3\}$	Reference	$d_{12} + \beta_{12}x_i$	$(d_{13} + \omega_{3,13}) + (\beta_{13} + \varphi_{3,13})x_i$

Table S2: Parameterisation of the DBT model that assess consistency of both the log odds ratio and the regression coefficient with independent treatment by average age interactions (*model 4.1a*) for the malaria and fabricated datasets.
DBT: design by treatment.
The parameterisation of the model that assesses consistency of the log odds ratio only (*model 4.2a*) is the same as that given in Table S2 but with the inconsistency parameter for the coefficient ($\varphi_{3,13}$) deleted; likewise the inconsistency parameter for the relative treatment effect ($\omega_{3,13}$) is omitted for the parameterisation of the model that assess consistency of the regression coefficient only (*model 4.3a*).

Model	Mean residual deviance	p_D	DIC	Between-site variance[1]	Mean of the exchangeable regression coefficients[1]	Variance of the exchangeable regression coefficients[1]
**Fixed-effect NMR model with independent interactions (*model 1a*)**	20.93	4.00	24.93	-	-	-
**Fixed-effect NMR model with exchangeable interactions (*model 1b*)**	20.94	4.00	24.95	-	0.0097 (-0.9439, 0.9663)	0.0142 (0.0000, 3.0370)
**Fixed-effect NMR model with common interactions (*model 1c*)**	22.29	3.00	25.29	-	-	-
**Random-effects NMR model with independent interactions (*model 1a*)**	18.68	7.45	26.13	0.0368 (0.0001, 0.3056)	-	-
**Random-effects NMR model with exchangeable interactions (*model 1b*)**	18.78	7.28	26.07	0.0334 (0.0001, 0.2993)	0.0119 (-0.9571, 0.9873)	0.0180 (0.0000, 3.0410)
**Random-effects NMR model with common interactions (*model 1c*)**	20.46	6.29	26.76	0.0273 (0.0001, 0.2809)	-	-

Table S3: Model fit assessment results and between-site variances for fixed-effect and random-effects NMR models for the malaria dataset.

Number of data points: 24

[1]Posterior median (95% credibility interval) presented.

DIC: deviance information criterion; NMR: network meta-regression.

	Posterior median (95% credibility interval)		
	**Artemether versus artesunate**	**Quinine versus artesunate**	**Quinine versus artemether**
**Fixed-effect NMR model with independent interactions (*model 1a*)**	0.0010 (-0.0181, 0.0203)	0.0146 (0.0032, 0.0261)	0.0136 (-0.0037, 0.0309)
**Fixed-effect NMR model with exchangeable interactions (*model 1b*)**	0.0022 (-0.0172, 0.0210)	0.0145 (0.0031, 0.0259)	0.0123 (-0.0040, 0.0301)
**Fixed-effect NMR model with common interactions (*model 1c*)**	0.0132 (0.0018, 0.0244)	0.0132 (0.0018, 0.0244)	Fixed at zero
**Random-effects NMR model with independent interactions (*model 1a*)**	0.0181 (-0.0027, 0.0466)	0.0167 (-0.0048, 0.0427)	0.0368 (0.0001, 0.3056)
**Random-effects NMR model with exchangeable interactions (*model 1b*)**	0.0031 (-0.0263, 0.0327)	0.0177 (-0.0028, 0.0459)	0.0150 (-0.0052, 0.0410)
**Random-effects NMR model with common interactions (*model 1c*)**	0.0150 (-0.0055, 0.0397)	0.0150 (-0.0055, 0.0397)	Fixed at zero

Table S4: Regression coefficients for the treatment by average age interaction from fixed-effect and random-effects NMR models for the malaria dataset.
NMR: network meta-regression.

Model	Mean residual deviance	p_D	DIC
**NMR model (*model 1c*)**	22.29	3.00	25.29
**DBT model assessing consistency of the log odds ratio and regression coefficient (*model 4.1c*)**	20.66	5.01	25.67
**DBT model assessing consistency of the log odds ratio only (*model 4.2c*)**	23.28	4.02	27.29
**DBT model assessing consistency of the regression coefficient only (*model 4.3c*)**	19.74	4.01	23.75

Table S5: Model fit assessment results for fixed-effect NMR and DBT models with common treatment by average age interactions for the malaria dataset.

Number of data points: 24

AR: artemether; AS: artesunate; DBT: design by treatment; DIC: deviance information criterion; QU: quinine; NMR: network meta-regression.

**Model**	**Parameter**	**Posterior median (95% credibility interval), P**				
		**AR vs. AS**	**QU vs. AS**	**AR vs. AS in design 1**	**QU vs. AS in design 2**	**Loop inconsistency for QU vs. AR (from design 3) compared with indirect evidence from AR vs. AS (from design 1) and QU vs. AS (from design 2)**
**NMR model (*model 1c*)**	**Log odds ratio (centred)**	0.2080 (-0.0441, 0.4592)	0.4350 (0.2923, 0.5772)	-	-	-
	**Regression coefficient for the interaction**	0.0132 (0.0018, 0.0244)	0.0132 (0.0018, 0.0244)	-	-	-
**DBT model assessing consistency of the log odds ratio and regression coefficient (*model 4.1c*)**	**Log odds ratio (centred)**	-	-	0.2493 (-0.3814, 0.8793)	0.4318 (0.2836, 0.5807)	0.1049 (-0.5969, 0.8097), P=0.77
	**Regression coefficient for the interaction**	-	-	0.0129 (0.0010, 0.0249)	0.0129 (0.0010, 0.0249)	0.0190 (-0.0006, 0.0388), P=0.06
**DBT model assessing consistency of the log odds ratio only (*model 4.2c*)**	**Log odds ratio (centred)**	-	-	0.2480 (-0.3871, 0.8874)	0.4319 (0.2833, 0.5793)	0.0515 (-0.6508, 0.7568), P=0.89
	**Regression coefficient for the interaction**	-	-	0.0129 (0.0010, 0.0248)	0.0129 (0.0010, 0.0248)	-
**DBT model assessing consistency of the regression coefficient only (*model 4.3c*)**	**Log odds ratio (centred)**	-	-	0.1638 (-0.0939, 0.4200)	0.4379 (0.2950, 0.5797)	-
	**Regression coefficient for the interaction**	-	-	0.0134 (0.0023, 0.0248)	0.0134 (0.0023, 0.0248)	0.0188 (-0.0007, 0.0385), P=0.06

Table S6: Results from fixed-effect NMR and DBT models with common treatment by average age interactions for the malaria dataset. AR: artemether; AS: artesunate; DBT: design by treatment; NMR: network meta-regression; P: probability of agreement between direct and indirect evidence; QU: quinine.

Dataset	Model	Mean residual deviance	p_D	DIC
**Dataset 1: No interaction and consistency**	**NMR model (*model 1a*)**	4.00	4.00	8.01
	**DBT model (*model 4.1a*)**	5.99	5.99	11.98
**Dataset 2: Interaction and consistency**	**NMR model (*model 1a*)**	4.00	4.00	8.00
	**DBT model (*model 4.1a*)**	5.99	5.99	11.97
**Dataset 3: Interaction and inconsistency**	**NMR model (*model 1a*)**	43.14	3.99	47.14
	**DBT model (*model 4.1a*)**	5.99	5.99	11.98
**Dataset 4: No interaction and inconsistency**	**NMR model (*model 1a*)**	184.36	4.00	188.36
	**DBT model (*model 4.1a*)**	6.00	6.00	12.00

Table S7: Model fit assessment results for fixed-effect NMR and DBT models assessing consistency of both the log odds ratio and regression coefficient with independent treatment by average age interactions for the fabricated datasets.

Number of data points: 30

AR: artemether; AS: artesunate; DBT: design by treatment; DIC: deviance information criterion; QU: quinine; NMR: network meta-regression.

ataset	Model	Parameter	Posterior median (95% credibility interval), P				
			**AR vs. AS**	**QU vs. AS**	**AR vs. AS in design 1**	**QU vs. AS in design 2**	**Loop inconsistency for QU vs. AR (from design 3) compared with indirect evidence from AR vs. AS (from design 1) and QU vs. AS (from design 2)**
**Dataset 1: No interaction and consistency**	**NMR model (*model 1a*)**	**Log odds ratio (uncentred)**	0.2002 (-0.0305, 0.4281)	0.2302 (0.0014, 0.4587)	-	-	-
		**Regression coefficient for the interaction**	0.0000 (-0.0090, 0.0091)	0.0000 (-0.0102, 0.0102)	-	-	-
	**DBT model (*model 4.1a*)**	**Log odds ratio (uncentred)**	-	-	0.1995 (-0.0947, 0.4946)	0.2298 (-0.0548, 0.5151)	-0.0001 (-0.2207, 0.2220), P=0.9996
		**Regression coefficient for the interaction**	-	-	0.0000 (-0.0108, 0.0108)	0.0000 (-0.0135, 0.0137)	0.0000 (-0.0208, 0.0209), P=0.9986
**Dataset 2: Interaction and consistency**	**NMR model (*model 1a*)**	**Log odds ratio (uncentred)**	0.2006 (-0.0539, 0.4514)	0.2302 (0.0043, 0.4558)	-	-	-
		**Regression coefficient for the interaction**	0.0200 (0.0074, 0.0327)	0.0200 (0.0080, 0.0321)	-	-	-
	**DBT model (*model 4.1a*)**	**Log odds ratio (uncentred)**	-	-	0.1997 (-0.1283, 0.5280)	0.2299 (-0.0255, 0.4863)	-0.0003 (-0.2291, 0.2313), P=0.9984
		**Regression coefficient for the interaction**	-	-	0.0200 (0.0049, 0.0351)	0.020 (0.0067, 0.0333)	-0.0001 (-0.0313, 0.0314), P=0.9970
**Dataset 3: Interaction and inconsistency**	**NMR model (*model 1a*)**	**Log odds ratio (uncentred)**	0.2081 (-0.0390, 0.4523)	0.1654 (-0.0503, 0.3808)	-	-	-
		**Regression coefficient for the interaction**	0.0187 (0.0082, 0.0292)	0.0335 (0.0244, 0.0425)	-	-	-
	**DBT model (*model 4.1a*)**	**Log odds ratio (uncentred)**	-	-	0.1997 (-0.1371, 0.5388)	0.2301 (-0.0196, 0.4792)	-0.6211 (-0.8385, -0.4017), P=0.0000
		**Regression coefficient for the interaction**	-	-	0.0100 (-0.0040, 0.0240)	0.0400 (0.0297, 0.0503)	-0.0300 (-0.0514, -0.0085), P=0.0060
**Dataset 4: No interaction and inconsistency**	**NMR model (*model 1a*)**	**Log odds ratio (uncentred)**	0.0877 (-0.1296, 0.3034)	0.3389 (0.1566, 0.5214)	-	-	-
		**Regression coefficient for the interaction**	-0.0098 (-0.0211, 0.0017)	-0.0001 (-0.0105, 0.0103)	-	-	-
	**DBT model (*model 4.1a*)**	**Log odds ratio (uncentred)**	-	-	0.1995 (-0.0916, 0.4917)	0.2297 (0.0231, 0.4367)	-1.3200 (-1.5420, -1.0950), P=0.0000
		**Regression coefficient for the interaction**	-	-	-0.0400 (-0.0553, -0.0247)	0.0400 (0.0272, 0.0529)	-0.0800 (-0.1031, -0.0569), P=0.0000

Table S8: Results from fixed-effect NMR and DBT models assessing consistency of both the log odds ratio and regression coefficient with independent treatment by average age interactions for the fabricated datasets.

AR: artemether; AS: artesunate; DBT: design by treatment; NMR: network meta-regression; P: probability of agreement between direct and indirect evidence; QU: quinine.

CODE FOR MODELS

R code for model 1a

```
library(R2WinBUGS)
library(coda)

working.directory="c:\\dir"
setwd(working.directory)
winbugs.directory="c:/Program Files/WinBUGS14/"

dat <- read.csv("data_design.csv")
x <- dat$x
logor <- dat$y
se <- dat$se
b <- dat$t1
t <- dat$t2
s <- dat$s
nt <- max(t,na.rm = TRUE)
N <- length(s)
mx=mean(x)
z=c(0,5,10,15,20,25,30,35, mx)
nz=length(z)
data= list("logor"=logor, "z"=z,  "nz"=nz, "se"=se,"t"=t, "b" = b, "nt" = nt, "N" = N, "x"=x,
"mx"=mx)

#initals
inits1 = list(d=c(NA,rep(0,(nt-1))), beta=c(NA,rep(0,(nt-1))))

#run model
model1a = bugs (data, inits1, model.file= "NMR_FE_LOR_IND.odc",
parameters.to.save=c("delta", "resdev2", "totresdev", "lorz",  "d", "dev",   "lor",  "betas",
"beta"), n.chains=1, n.iter=300000, n.burnin=100000, n.thin=5,codaPkg=FALSE,
bugs.directory=winbugs.directory, working.directory=working.directory)
```

Winbugs code for model 1a (file called "NMR_FE_LOR_IND.odc")

```
model{
for(i in 1:N){
	prec[i] <- pow(se[i],-2)
	logor[i] ~ dnorm(delta[i], prec[i])
	delta[i]<- (d[t[i]] - d[b[i]]) + (beta[t[i]]-beta[b[i]]) * (x[i]-mx)
	dev[i] <-logor[i]-delta[i]
	resdev2[i]<-(dev[i]*dev[i])/(se[i]*se[i])
		}

totresdev<-sum(resdev2[])

d[1]<-0
beta[1] <- 0
```

```
for (k in 2:nt){
       d[k] ~ dnorm(0,0.00001)
       beta[k]~dnorm(0,0.00001)
                      }

for (k in 1:nt){
     for (j in 1:nz) {
          dz[j,k] <- d[k] - (beta[k])*(mx-z[j])
                      }
                           }

for (c in 1:(nt-1)) {
    for (k in (c+1):nt) {
         betas[c,k] <- beta[k] - beta[c]
         lor[c,k] <- (d[k]-d[c])

                 for (j in 1:nz) {
                      lorz[j,c,k] <- (dz[j,k]-dz[j,c])
                                   }
                             }
                       }
       }
```

<u>CSV file (file called "data_design.csv")</u>

s is the study number, t1 is the treatment in arm 1, t2 is the treatment in arm 2, y is the log odds ratio, se is its standard error, x is the covariate age, and design is the design group.

s	t1	t2	y	se	x	design
1	1	2	0.69	0.361414	32.25	1
2	1	2	-0.15	0.58266	27.33333	1
3	1	3	1.38	0.876554	34.47	2
4	1	3	1.03	0.453038	30.95	2
5	1	3	0.32	0.716684	5.5	2
6	1	3	0.52	0.137157	27.9	2
7	1	3	0.28	0.092367	2.85	2
8	1	3	0.72	1.250403	4.5	2
9	1	3	0.64	0.639465	28.5	2
10	1	3	0.75	0.518875	25	2
11	2	3	1.1	1.662597	3.845	3
12	2	3	-0.23	0.601291	3.5	3
13	2	3	0.35	0.239694	30	3
14	2	3	0.24	0.693575	6.2	3
15	2	3	2.23	1.191545	31.05	3
16	2	3	1.43	0.525306	26.5	3
17	2	3	-0.79	0.903179	6.8	3
18	2	3	-0.69	0.458817	2.333333	3

19	2	3	0.69	1.271868	3.93	3
20	2	3	0.45	0.46278	3.15	3
21	2	3	0	2.030077	7	3
22	2	3	0.45	0.465031	3.075	3
23	2	3	0.06	0.204621	3.916667	3
24	2	3	0.65	0.76742	3	3

R code for model 2.1a

```
library(R2WinBUGS)
library(coda)

working.directory="c:\\dir"
setwd(working.directory)
winbugs.directory="c:/Program Files/WinBUGS14/"

splitfn <- function(pair, N, b, t){
split<-rep(0,N)
for(i in 1:N){
if (b[i]==pair[1] & t[i]==pair[2]){
    split[i] <- 1
                                    }
            }
out=cbind(split, b, t)
out
}

dat <- read.csv("data_design.csv")
x <- dat$x
logor <- dat$y
se <- dat$se
b <- dat$t1
t <- dat$t2
s <- dat$s
nt <- max(t,na.rm = TRUE)
N <- length(s)
mx=mean(x)

#initials
inits1 = list(direct=0,d=c(NA,rep(0,(nt-1))), directbeta=0,beta=c(NA,rep(0,(nt-1))))

# Define node to split: (1,2)
pair <- c(1,2)
splitx=splitfn(pair, N, b, t)
split <- splitx[,1]
bugs.data(list("logor"=logor,"se"=se,"t"=t,"b" = b, "nt" = nt, "N" = N, "split" = split, "pair" =
pair , "x"=x, "mx"=mx  ) )
```

```
model21a=bugs(data = "data.txt", inits = inits1, parameters.to.save = c("delta", "resdev2",
"direct", "d", "lor",   "prob",  "dev", "diff",  "directbeta",  "diffbeta", "probbeta", "betas",
"beta"), model.file = "NMR_FE_LOR_IND_SPLIT.odc", n.chains = 1, n.iter = 300000,
n.burnin = 100000, n.thin = 5, bugs.directory = winbugs.directory,
working.directory=working.directory)

#repeat for other nodes
```

Winbugs code for model 2.1a (file called "NMR_FE_LOR_IND_SPLIT.odc")

```
model {
for(i in 1:N){
    prec[i] <- pow(se[i],-2)
    logor[i] ~ dnorm(delta[i], prec[i])
    index[i] <- split[i] * (equals(b[i], pair[1])) * (equals(t[i], pair[2]))
    delta[i] <-  deltat[i] + deltab[i]
    deltat[i]<- (d[t[i]] - d[b[i]])*(1-index[i]) + direct*index[i]
    deltab[i]<- ((beta[t[i]]-beta[b[i]]) * (x[i]-mx))*(1-index[i]) + directbeta*(x[i]-
mx)*index[i]
    dev[i] <-logor[i]-delta[i]
    resdev2[i]<-(dev[i]*dev[i])/(se[i]*se[i])
              }

totresdev<-sum(resdev2[])

direct ~ dnorm(0,0.00001)
directbeta ~ dnorm(0,0.00001)
d[1] <- 0
beta[1] <- 0

for (k in 2:nt) {
    d[k] ~ dnorm(0,0.00001)
    beta[k] ~ dnorm(0,0.00001)
                 }

for (c in 1:(nt-1)) {
   for (k in (c+1):nt)  {
         betas[c,k] <- beta[k] - beta[c]
          lor[c,k] <- (d[k]-d[c])
                        }
                    }

diff <- direct - lor[pair[1], pair[2]]
prob <- step(diff)
diffbeta <- directbeta - betas[pair[1], pair[2]]
probbeta <- step(diffbeta)
}
```

R code for model 3.1a

```
library(R2WinBUGS)
library(coda)

working.directory="c:\\dir"
setwd(working.directory)
winbugs.directory="c:/Program Files/WinBUGS14/"


dat <- read.csv("data_design.csv")
x <- dat$x
logor <- dat$y
se <- dat$se
b <- dat$t1
t <- dat$t2
s <- dat$s
nt <- max(t,na.rm = TRUE)
N <- length(s)
mx=mean(x)
data= list("logor"=logor,  "se"=se,"t"=t, "b" = b, "nt" = nt, "N" = N, "x"=x, "mx"=mx)

#initals
inits1 = list(d= structure(.Data= c(NA,NA,NA,   0,NA,NA,   0,0,NA),.Dim = c(3,3)), beta=
structure(.Data= c(NA,NA,NA,   0,NA,NA,   0,0,NA),.Dim = c(3,3)))

#run model
model41a = bugs (data, inits1, model.file= "NMA_FE_LOR_IND_URM.odc",
parameters.to.save= c("delta", "resdev2",    "d", "totresdev",  "dev",  "beta"),
n.chains=1, n.iter=300000, n.burnin=100000, n.thin=5,codaPkg=FALSE,
bugs.directory=winbugs.directory, working.directory=working.directory)
```

<u>Winbugs code for model 3.1a (file called “NMR_FE_LOR_IND_URM.odc”)</u>

```
model{
for(i in 1:N){
     prec[i] <- pow(se[i],-2)
     logor[i] ~ dnorm(delta[i], prec[i])
     delta[i]<- (d[b[i],t[i]]) + (beta[b[i],t[i]]) * (x[i]-mx)
     dev[i] <-logor[i]-delta[i]
     resdev2[i]<-(dev[i]*dev[i])/(se[i]*se[i])
}

totresdev<-sum(resdev2[])

for (k in 1:nt) {
   d[k,k] <- 0
   beta[k,k] <- 0
                  }

for (c in 1:(nt-1)){
    for (k in (c+1):nt) {
```

```
                d[c,k] ~ dnorm(0,0.00001)
                beta[c,k]~dnorm(0,0.00001)
                                }
                        }
}
```

__R code for model 4.1a__

```
library(R2WinBUGS)
library(coda)

working.directory="c:\\dir"
setwd(working.directory)
winbugs.directory="c:/Program Files/WinBUGS14/"

dat <- read.csv("data_design.csv")
x <- dat$x
logor <- dat$y
se <- dat$se
b <- dat$t1
t <- dat$t2
s <- dat$s
design <- dat$design
nt <- max(t,na.rm = TRUE)
N <- length(s)
mx=mean(x)
data= list("logor"=logor, "se"=se,"t"=t, "b" = b, "nt" = nt, "N" = N, "x"=x, "mx"=mx,
"design"=design)

#initals
inits1 = list(d=c(NA,rep(0,(nt-1))),beta=c(NA,rep(0,(nt-1))),incons = structure(.Data =
c(NA,NA, NA, NA,NA,NA, NA,  NA, 0),.Dim = c(3,3)),inconsbeta  = structure(.Data =
c(NA,NA, NA, NA,NA,NA, NA,  NA, 0),.Dim = c(3,3)))

#run model
model31a = bugs (data, init1, model.file= "NMR_FE_LOR_IND_TBD.odc",
parameters.to.save= c("delta", "resdev2", "d", "dev", "lor", "incons",
"prob1","inconsbeta","totresdev","beta","betas","probbeta1" ),
n.chains=1, n.iter=300000, n.burnin=100000, n.thin=5,codaPkg=FALSE,
bugs.directory=winbugs.directory,
working.directory=working.directory)
```

__Winbugs code for model 4.1a (file called “NMR_FE_LOR_IND_TBD.odc”)__

```
model{
for(i in 1:2){
     for(j in 1:2){
          incons[i,j] <- 0
          inconsbeta[i,j] <- 0
```

```
            }
        }
incons[3,1] <- 0
incons[3,2] ~ dnorm(0,0.00001)
inconsbeta[3,1] <- 0
inconsbeta[3,2] ~ dnorm(0,0.00001)

for(i in 1:N){
   prec[i] <- pow(se[i],-2)
   logor[i] ~ dnorm(delta[i], prec[i])
   delta[i]<- (d[t[i]] - d[b[i]]) + ((beta[t[i]]-beta[b[i]]) * (x[i]-mx)) + incons[design[i],
mlong[i]]   + ((x[i]-mx) * inconsbeta[design[i], mlong[i]])
   dev[i] <-logor[i]-delta[i]
   resdev2[i]<-(dev[i]*dev[i])/(se[i]*se[i])
        }

totresdev<-sum(resdev2[])

d[1]<-0
beta[1] <- 0

for (k in 2:nt){
  d[k] ~ dnorm(0,0.00001)
  beta[k]~dnorm(0,0.00001)
          }

for (c in 1:(nt-1)) {
  for (k in (c+1):nt)  {
       betas[c,k] <- beta[k] - beta[c]
        lor[c,k] <- (d[k]-d[c])
               }
           }

prob1 <- step(incons[3,2])
probbeta1 <- step(inconsbeta[3,2])
}
```

Example R code to produce the fabricated datasets

Dataset 1: No interaction and consistency

```
library(truncnorm)
library(xlsx)

NS=30
NC=3
s=c(1:30)

#set up treatments
```

```
t1=c(rep(1, NS/NC), rep(1, NS/NC), rep(2, NS/NC))
t2=c(rep(2, NS/NC), rep(3, NS/NC), rep(3, NS/NC))

#simulate covariate data
xab=rtruncnorm(n=10, a=0, mean=17, sd=10)
xac=rtruncnorm(n=10, a=0, mean=17, sd=10)
xbc=rtruncnorm(n=10, a=0, mean=17, sd=10)
x=c(xab,xac,xbc)

#simulate outcome data
dab=0.20
dac=0.23
dbc=dac-dab
bab=0
bac=0
bbc=bac-bab

yab=rep(NA,(NS/NC))
yac=rep(NA,(NS/NC))
ybc=rep(NA,(NS/NC))

for(i in 1:(NS/NC)){
yab[i]=dab + xab[i]*bab
yac[i]=dac + xac[i]*bac
ybc[i]=dbc + xbc[i]*bbc
}

y=c(yab,yac,ybc)

#set up variances
se=c(rep(0.2,(NS/NC)), rep(0.2,(NS/NC)),rep(0.2,(NS/NC)))

data=cbind(x,s,t1,t2, y, se)
write.csv(data, "c:/dirx/noint_dbconsistent.csv")
```
